# *SLC27A2* mediates FAO in colorectal cancer through nongenic crosstalk regulation of the *PPARs* pathway

**DOI:** 10.1186/s12885-023-10816-3

**Published:** 2023-04-11

**Authors:** Kun Shang, Nina Ma, Juanjuan Che, Huihui Li, Jiexuan Hu, Haolin Sun, Bangwei Cao

**Affiliations:** Department of Oncology, Beijing Friendship Hospital, Capital Medical University, 95 Yongan Road, Xi-Cheng District, Beijing, 100050 China

**Keywords:** *SLC27A2*, Colorectal cancer, FAO, *PPARs*

## Abstract

**Background:**

Peroxisome proliferator activated receptors (*PPARs*) are a nuclear hormone receptors superfamily that is closely related to fatty acid (FA) metabolism and tumor progression. Solute carrier family 27 member 2 (*SLC27A2*) is important for FA transportation and metabolism and is related to cancer progression. This study aims to explore the mechanisms of how *PPARs* and *SLC27A2* regulate FA metabolism in colorectal cancer (CRC) and find new strategies for CRC treatment.

**Methods:**

Biological information analysis was applied to detect the expression and the correlation of *PPARs* and *SLC27A2* in CRC. The protein–protein interaction (PPI) interaction networks were explored by using the STRING database. Uptake experiments and immunofluorescence staining were used to analyse the function and number of peroxisomes and colocalization of FA with peroxisomes, respectively. Western blotting and qRT‒PCR were performed to explore the mechanisms.

**Results:**

*SLC27A2* was overexpressed in CRC. *PPARs* had different expression levels, and *PPARG* was significantly highly expressed in CRC. *SLC27A2* was correlated with *PPARs* in CRC. Both *SLC27A2* and *PPARs* were closely related to fatty acid oxidation (FAO)‒related genes. *SLC27A2* affected the activity of ATP Binding Cassette Subfamily D Member 3 (*ABCD3*), also named *PMP70*, the most abundant peroxisomal membrane protein. We found that the ratios of *p-Erk/Erk* and *p-GSK3β/GSK3β* were elevated through nongenic crosstalk regulation of the *PPARs* pathway.

**Conclusions:**

*SLC27A2* mediates FA uptake and beta-oxidation through nongenic crosstalk regulation of the *PPARs* pathway in CRC. Targeting *SLC27A2/FATP2* or *PPARs* may provide new insights for antitumour strategies.

**Supplementary Information:**

The online version contains supplementary material available at 10.1186/s12885-023-10816-3.

## Background

Colorectal cancer (CRC), ranking as the third most frequent cancer and second leading cause of cancer‒related deaths, is a major challenge in healthcare worldwide [1]. Most patients are diagnosed with advanced or metastatic disease [2]. The proportion of patients younger than 50 years is rising, owing to hereditary or environmental risk factors [3]. Precise management of CRC, involving single or combined reagents, is needed [4]. Metabolic reprogramming of tumor and the tumor microenvironment (TME), including cells, cytokines, nutrients or metabolites, supports proliferation, migration, immune escape or resistance in cancers [5, 6].

Lipid metabolic hallmarks play a pivotal role in CRC. Fatty acids (FAs) as the essential molecules of lipid can maintain membrane homeostasis, and regulate cell signalling and energy metabolism [7]. FA can be taken up from the TME or de novo synthesized in cells. FA uptake occurs through transmembrane proteins, including fatty acid translocase (*FAT, CD36*), fatty acid binding proteins (*FABPs*) and fatty acid transport proteins (*FATPs, SLC27s*) [8]. The intracellular FA pool is the source of cell structural molecules and metabolism. Fatty acid beta-oxidation (FAO) is an important metabolic process in mitochondria or peroxisome [9]. For tumor progression, cancer cells reprogram to FAO to produce ATP more efficiently. Medium-chain FAs are mainly catabolized in mitochondria and long-chain or very long-chain FAs are primarily catabolized in peroxisome [10].

Peroxisome proliferator activated receptors (*PPARs*) are nuclear hormone receptors (NHR) and are ligand-dependent transcriptional regulators. Recent researches have revealed that *PPAR* agonists or antagonists can regulate cell metabolism, including the FAO process, exhibiting anticancer effects [11]. Recently, studies about single-cell genomic and transcriptomic landscapes of metastatic colorectal cancer (mCRC) patients and patient-derived tumor organoids have revealed that the *PPAR* signaling pathway was aberrantly activated in mCRC. *PPAR* inhibitors can suppress the proliferation and promote the apoptosis of CRC organoids, indicating it’s critical role in mCRC tumorigenesis [12]. The *PPAR* pathway can regulate FAO to induce tumorigenesis in intestinal stem cells (ISCs) in high-fat diet (HFD) feeding mouse models [13, 14]. Ligand-activated *PPARs* heterodimerize with retinoid X receptor (RXR) and bind to specific DNA response elements (PPREs), regulating lipids homeostasis and metabolism [15]. In previous studies, solute carrier family 27 member 2 (*SLC27A2*) was reported to regulate cancer proliferation, metastasis, inflammation and immunosuppression [16, 17]. We investigated the expression of *SLC27A2* and *PPARs* aiming to find new metabolic therapies for CRC.

## Materials and methods

### Cell culture and transient transfection

The human colorectal cancer cell lines, HCT-15 and SW480 were purchased from ATCC and passed short tandem repeat (STR) detection. The cells were cultured in RPMI 1640 medium (CORNIN, USA) containing 10% fetal bovine serum (FBS, Gibco, USA), at 37 °C and 5% CO_2_. For further study, we cultured cells with palmitic acid (PA, 100 uM, Sigma, dissolved in DMSO, NaOH, BSA) and generated the fatty cells, in which lipid droplet accumulated in the cytosol and Oil-Red-O (ORO) staining were observed [18]. Transient transfections were conducted by MegaTran 2.0 or siTran 2.0 to overexpress or knock down *SLC27A2* expression, according to the manufacturer’s protocol, respectively. And qRT‒PCR was used to test the efficacy. In our previous study we used two siRNAs to knock down *SLC27A2* and the efficacy was tested by qRT‒PCR and western blotting. The sequence of siRNA-*SLC27A2*-3 in this study was 5'-CGACAGAGUUGGAGAUACATT- 3'.

### Western blot

Whole proteins were extracted by cell lysis buffer (Beyotime, P0013B, Shang hai) and quantified by a BCA protein assay kit (Thermo Fisher, 23,227, USA). Proteins were separated by a 10% SDS‒PAGE gel and transferred to a PVDF membrane. Then, the PVDF membrane was blocked with 5% nonfat milk for 2 h at room temperature (RT), and incubated with primary antibodies overnight (4 °C) and secondary HRP-conjugated antibodies for 2 h (RT), respectively. The PVDF membrane was washed with Tris buffered Saline with Tween-20 (TBST) buffer after every step. The blots were cut prior to hybridisation with antibodies. Western Blot Stripping Buffer (Bioss, C05-03,041) was used for breaking antibody-antigen interactions to detect multiple target protein by using different antibodies. The molecular weights of target proteins are very similar and we cropped the blots closely. The original gels and multiple exposure images was shown in Supplementary Fig. 1. Blots were detected by an enhanced chemiluminescence system (Bio-Rad, USA). The relative gray value was measured by Image J. We performed three independent repetitions of the experiments for each dataset. The specific primary antibodies as follows: anti-β-actin (42KD, 1:5000, 20,536–1-AP, Proteintech), anti-SLC27A2 (70KD, 1:2000, 14,048–1-AP, Proteintech), anti-PPARG (50KD, 1:2000, 16,643–1-AP, Proteintech), anti-Erk1/2 (42/44KD, 1:1000, 11,257–1-AP, Proteintech), anti-p-Erk1/2 (42/44KD, 1:2000, 28,733–1-AP, Proteintech), anti-GSK3β(47KD, 1:500, ET1607-71, HUABIO), and anti-p-GSK3β (47KD, 1:500, ET1607-60, HUABIO).

### RNA extraction and quantitative reverse transcriptase-PCR (qRT‒PCR)

Total RNA was extracted by using TRIzol Reagent (Invitrogen, 15,596–018, USA). All steps were performed according to the manufacturer’s instructions. RNA concentrations were quantified with a Nanodrop 2000 system (Thermo Fisher Scientific, USA), and cDNAs were obtained with a reverse transcriptase kit (TaKaRa, Japan). qRT‒PCR was performed by SYBR Premix ExTaqTM II (Takara, Japan). The mRNA levels were normalized to β-actin and 2^−ΔΔ^Ct was calculated for analysis. The mRNA expression average three different experiments. The primers used in qRT‒PCR are listed in Table S1.

### Immunofluorescence staining and FA uptake

HCT-15 and SW480 cells were digested, collected and counted after transfection for 24 h. A total of 2 ~ 2.5 × 10^5^ cells were seeded in 6-well culture plates per well, in which a slide had been placed. After attaching, the cells were cocultured with fluorescent BODIPYTM FL C16 fatty acid (24 h, 10 μM, Invitrogen, D3821, USA), which is a fluorescence labelled palmitic acid (PA). Slides with cells were collected, washed gently with PBS, fixed in 4% PFA for 15 min, incubated in 0.25% Triton X-100 for 10 min to rupture the cell membranes, and blocked for 1 h by using PBST (1% BSA). The cells were incubated in anti-PMP70 (1: 500, Abcam, ab3421) primary antibody (overnight, 4 °C). Next, the slides were washed with PBS and incubated with fluorescent secondary antibody (Alexa Fluor 568 goat anti-rabbit IgG, 1:100, Life Technologies, Waltham, MA USA) in the dark (1 h, RT). Finally, the slides were washed and stained with DAPI (sc-24941, Santa Cruz Biotechnology, Dallas, TX, USA) and imaged by using a confocal microscope (Olympus, IX83, FLUOVIEW FV1200, Tokyo, Japan). The relative fluorescence intensity was measured by Image J. We performed three independent repetitions of the experiments.

### Biological information analysis

Extensive RNA sequencing data from The Cancer Genome Atlas (TCGA) and the Genotype-Tissue Expression (GTEx) databases were collected in the Gene Expression Profiling Interactive Analysis (GEPIA) database (http://gepia.cancer-pku.cn/) [19]. In our study, we explored the expression of *SLC27A2* and *PPARs* in CRC through the GEPIA database (http://gepia2.cancer-pku.cn/#analysis) by ‘GEPIA2 Expression DIY on Box Plot’ mode. We explored the correlation of ‘*SLC27A2* with *PPARs*’ and ‘*SLC27A2* with FAO‒related genes’ in CRC through the GEPIA database by the ‘GEPIA2 Correlation Analysis’ mode. The protein–protein interaction (PPI) networks were analyzed on the STRING database Version 11.5 (https://cn.string-db.org/) [20]. The PPI networks of *SLC27A2/PPARG* in CRC were visualized by using the following steps: ‘STRING Protein by name’, ‘Organisms Homo sapiens’, and ‘Viewers by Network’. The graphic abstract was generated by the FigDraw database (https://www.figdraw.com/static/index.html#/, accession numbers: 788566346027118592; Figure ID:UWYWTc1d3c).

### Statistical analysis

GraphPad Prism 5.0 software was used for statistical analysis. Data were expressed as the mean ± SEM. The differences between two groups were analyzed by Student’s t test, and three groups or more were analyzed by one-way ANOVA. *P* < 0.05 was considered statistically significant. *P < 0.05; **P < 0.01; ***P < 0.001.

## Results

### SLC27A2 was related to PPARs in colorectal cancer

**Fig. 1:**
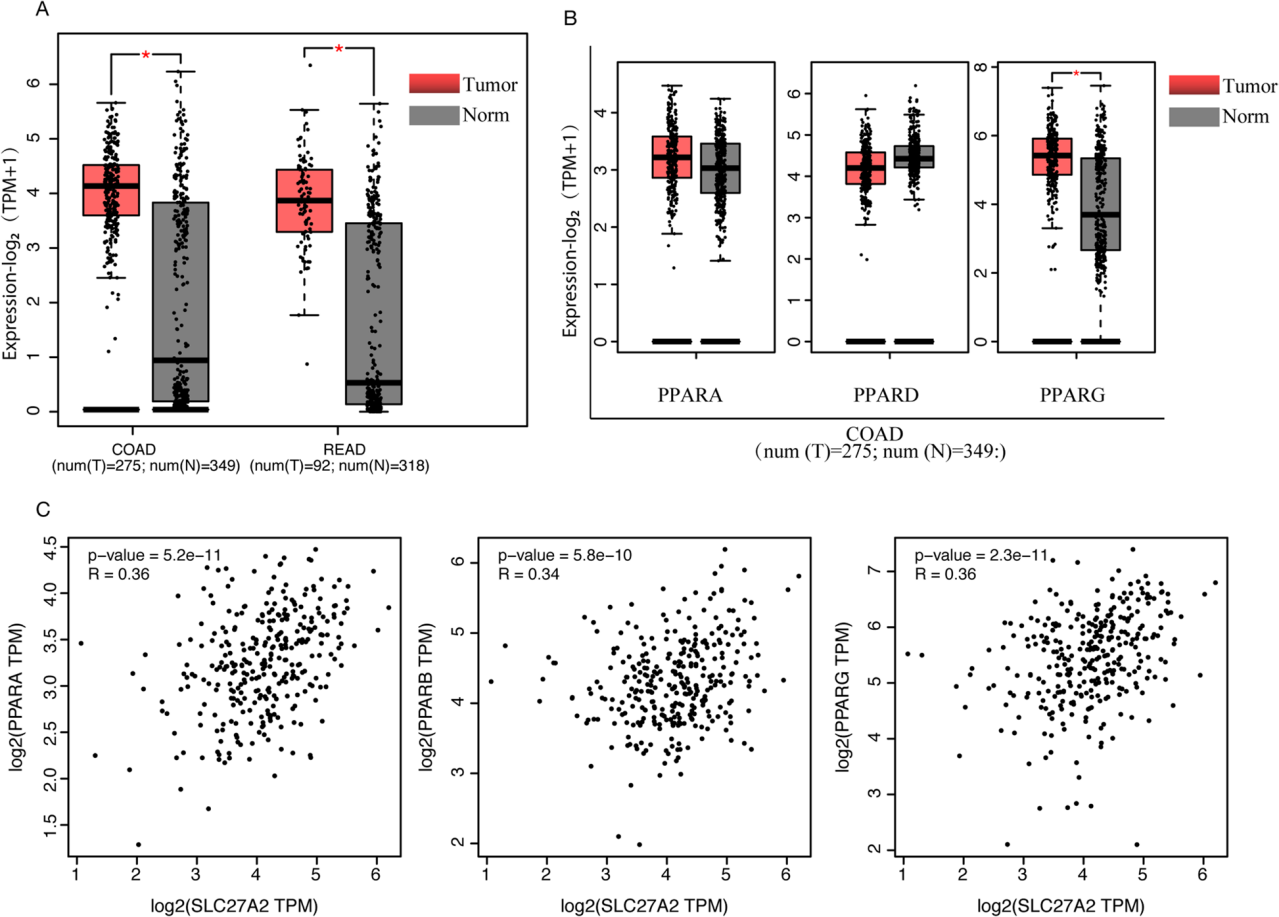
SLC27A2 was related to PPARs in colorectal cancer.** A** SLC27A2 was overexpressed in CRC from (GEPIA) database. **B** PPARs had different expressions in colorectal cancer, and PPARG was significantly highly expressed in CRC from (GEPIA) database. **C** SLC27A2 had a relationship with PPARs in CRC from GEPIA database. *P < 0.05; **P < 0.01; ***P < 0.001*.* T: tumor; N: normal; COAD: colon adenocarcinoma; READ: rectum adenocarcinoma; SLC27A2: Solute carrier family 27 member 2; PPARA: Peroxisome proliferator activated receptor A; PPARD: Peroxisome proliferator activated receptor D; PPARG: Peroxisome proliferator activated receptor G

*SLC27A2* is a protein–coding gene, and the encoded protein *FATP2* acts as a transporter to take up FAs or an isozyme to convert long-chain fatty acids into fatty acyl-CoA [21]. Studies have shown that *SLC27A2* is elevated in cancers and promotes cancer progression [16]. We explored the GEPIA database and found that *SLC27A2* was overexpressed in CRC compared to para-normal tissues (Fig. 1A). Our previous experiments elucidated that overexpression or knockdown of *SLC27A2* could promote or suppress CRC cells proliferation, cell cycle or migration. *PPARs* were also expressed differently in CRC, and *PPARG* was highly expressed with statistical significance (Fig. 1B). *PPARs* are pivotal factors in regulating lipid metabolism. We performed correlation analysis between *SLC27A2* and *PPARs* and the protein–protein interaction (PPI) networks showed that *SLC27A2* was related to *PPARs* in CRC (Fig. 1C). The original data was provided in Supplementary Fig. 2.

### SLC27A2 was associated with the PPARs pathway

**Fig. 2:**
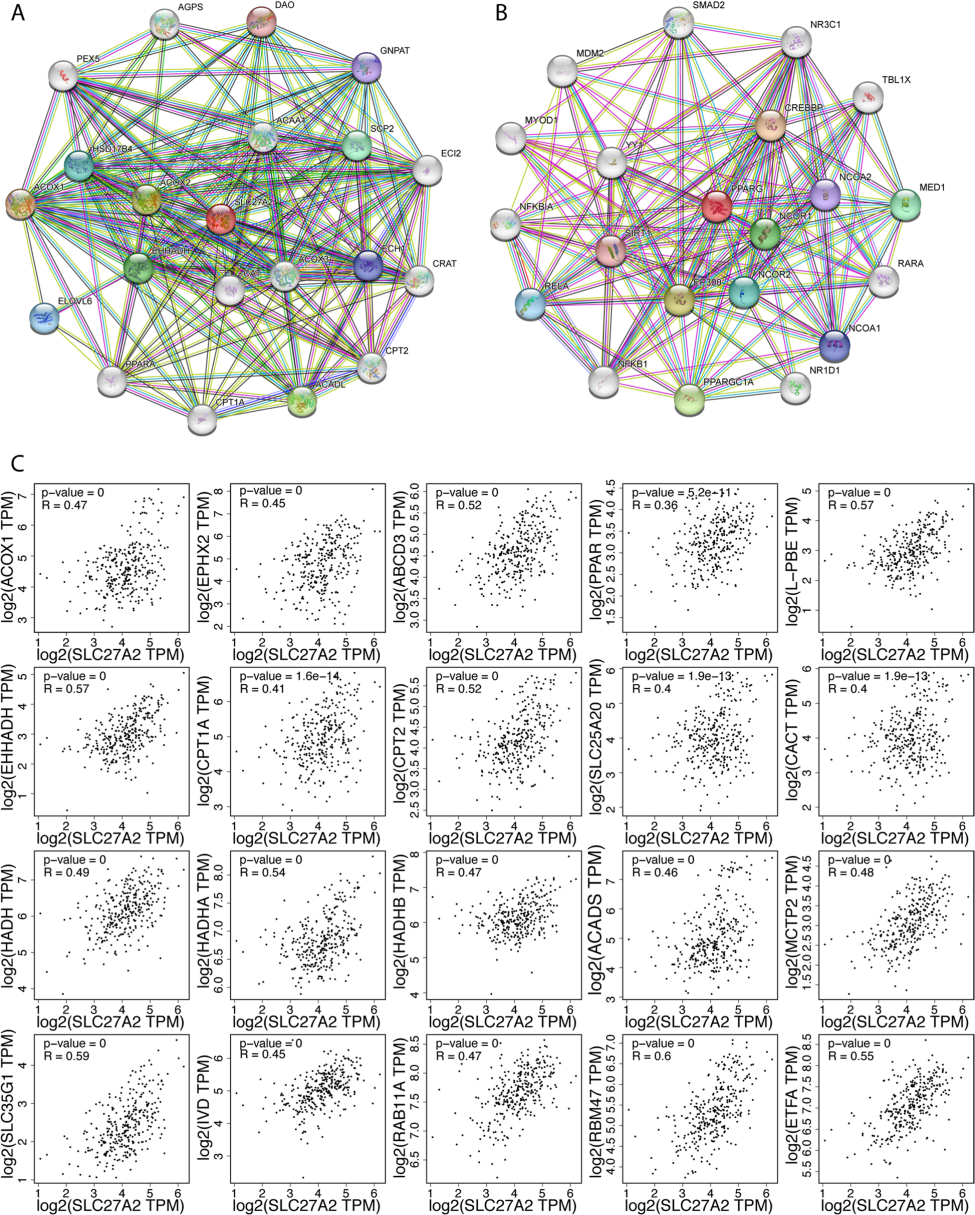
SLC27A2 was associated with the PPARs pathway.** A** SLC27A2 correlated with genes encoding proteins associated with FAO metabolism on the STRING database. **B** The correlated proteins with PPARG in CRC from the STRING database. **C** SLC27A2 had a close relationship with FAO metabolic genes in CRC, both in mitochondria and peroxisomes on the STRING database

We explored the protein–protein interaction (PPI) networks between proteins on the STRING database (https://cn.string-db.org/). The results showed that *SLC27A2* was correlated with FAO metabolic genes, whether in mitochondria or peroxisome (Fig. 2A). *PPARG* is a nuclear hormone receptor (NHR), that is mainly located in the nucleus, cytosol, or peroxisome. Additionally, *PPARG* is involved in energy metabolism. As a ligand–inducible transcriptional regulator, *PPARG* can be activated by FA. We investigated the proteins correlated with *PPARG* in the STRING database and found that it had a relationship with other NHRs (Fig. 2B). Through gene correlation analysis in the GEPIA database (http://gepia.cancer-pku.cn/), we revealed that the expression level of *SLC27A2* was significantly correlated with FAO metabolic genes (Fig. 2C).

### SLC27A2 was associated with FAO metabolic genes in colorectal cancer

**Fig. 3:**
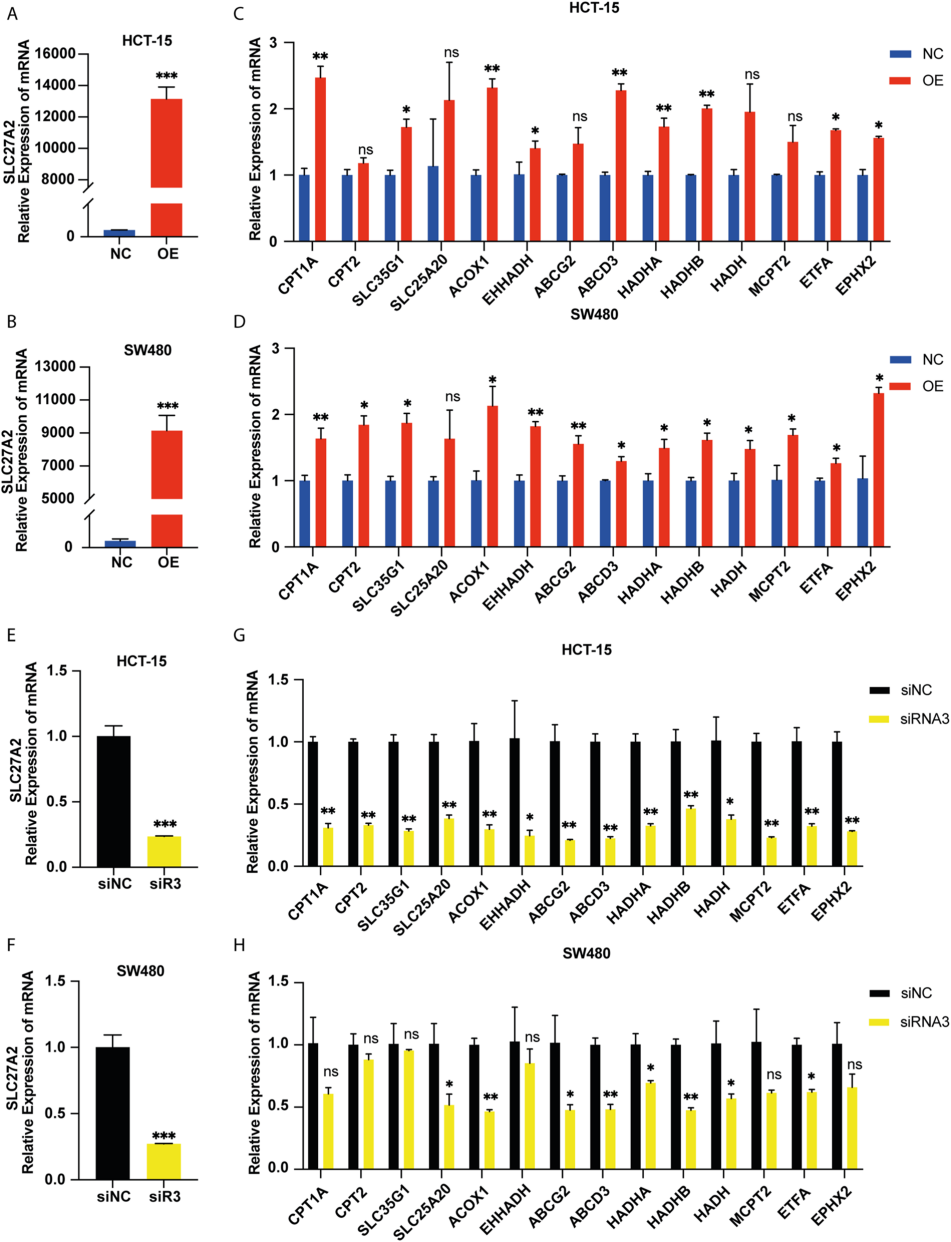
SLC27A2 was associated with FAO metabolic genes in colorectal cancer.** A** ~ **B** SLC27A2 was overexpressed in HCT-15 and SW480 by plasmids and tested by qRT‒PCR. **C** ~ **D** The expression of FAO related genes were measured when SLC27A2 was overexpressed in CRC and the mRNA expression of CPT1A, SLC35G1, ACOX1, EHHADH, ABCD3, HADHA, HADHB, ETFA, and EPHX2 elevated in HCT-15 and SW480. **E** ~ **F** SLC27A2 was knocked down in HCT-15 and SW480 by siRNAs and verified using qRT‒PCR. **G** ~ **H** The mRNA expression levels of FAO–related genes were measured when SLC27A2 was knocked down in CRC and the mRNA expression of SLC25A20, ACOX1, ABCG2, ABCD3, HADHA, HADHB, HADH, and ETFA decreased in HCT-15 and SW480. NC: negative control; OE: over expression

To investigate the influence of *SLC27A2* on FAO–related gene expression, we overexpressed *SLC27A2* by plasmids, and the efficiency was tested by qRT–PCR (Fig. 3A ~ B). We extracted RNA from HCT-15 and SW480 cells transfected with plasmids and demonstrated that the mRNA levels of FAO–related genes were increased (Fig. 3C ~ D). Similarly, we knocked down *SLC27A2* by siRNAs (Fig. 3E ~ F) and the mRNA levels of FAO–related genes concomitantly decreased (Fig. 3G ~ H).

### SLC27A2 regulated the function and number of peroxisomes in colorectal cancer

**Fig. 4:**
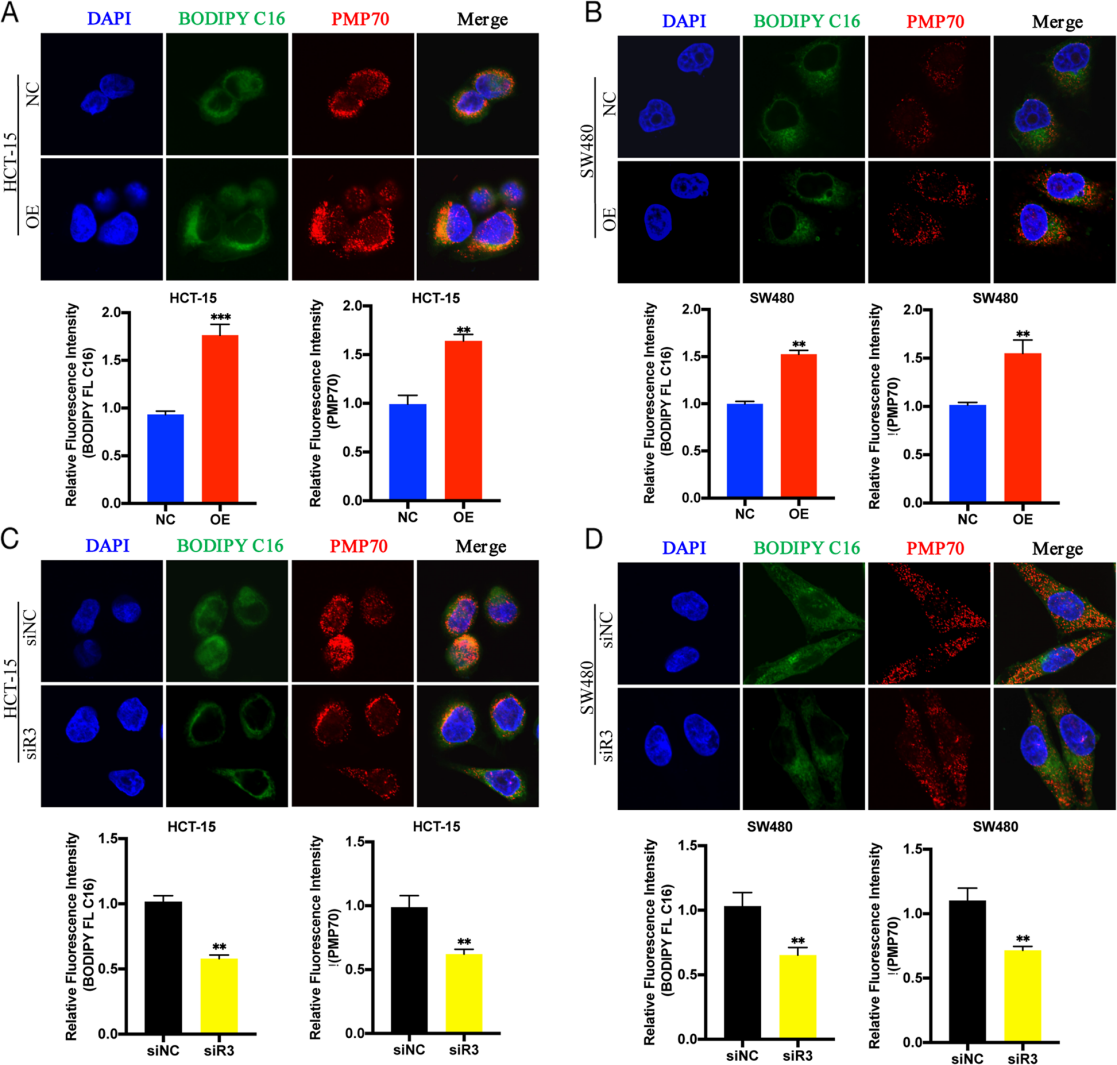
SLC27A2 regulated the function and number of peroxisomes in colorectal cancer.** A** ~ **B** The function and number of peroxisomes were enhanced, and the uptake levels of fluorescent FA (BODIPY FL C16, PA, 10 μM) were elevated and the colocalization of FA with peroxisomes were enhanced when SLC27A2 was overexpressed in CRC. **C** ~ **D** The function and number of peroxisomes, uptake level of fluorescent FA, and colocalization diminished when SLC27A2 was knocked down in CRC. NC: negative control; OE: over expression; PMP70: belonged to the superfamily of ATP-binding cassette (ABC) transporters, also named ABCD3 (ATP Binding Cassette Subfamily D Member 3)

*ABCD3* (ATP Binding Cassette Subfamily D Member 3), also named *PMP70*, belongs to the superfamily of ATP-binding cassette (ABC) transporters. Peroxisomal ABC transporters are involved in lipid metabolism and the PPARs pathway, particularly in FAO metabolism [22]. The expression level of *ABCD3* increased or decreased upon *SLC27A2* overexpression or knockdown, respectively (Fig. 3). We conducted coculture experiments and found that FA uptake levels were elevated when *SLC27A2* was overexpressed. The *PMP70* level, which represents the number and function of peroxisomes [23], increased in the overexpression group. Interestingly, fluorescence–labelled FAs colocalized with *PMP70* (Fig. 4A ~ B). Similar results were observed when *SLC27A2* was knocked down (Fig. 4C ~ D).

### SLC27A2 reprogrammed colorectal cancer non-genic crosstalk regulation of PPARG

**Fig. 5:**
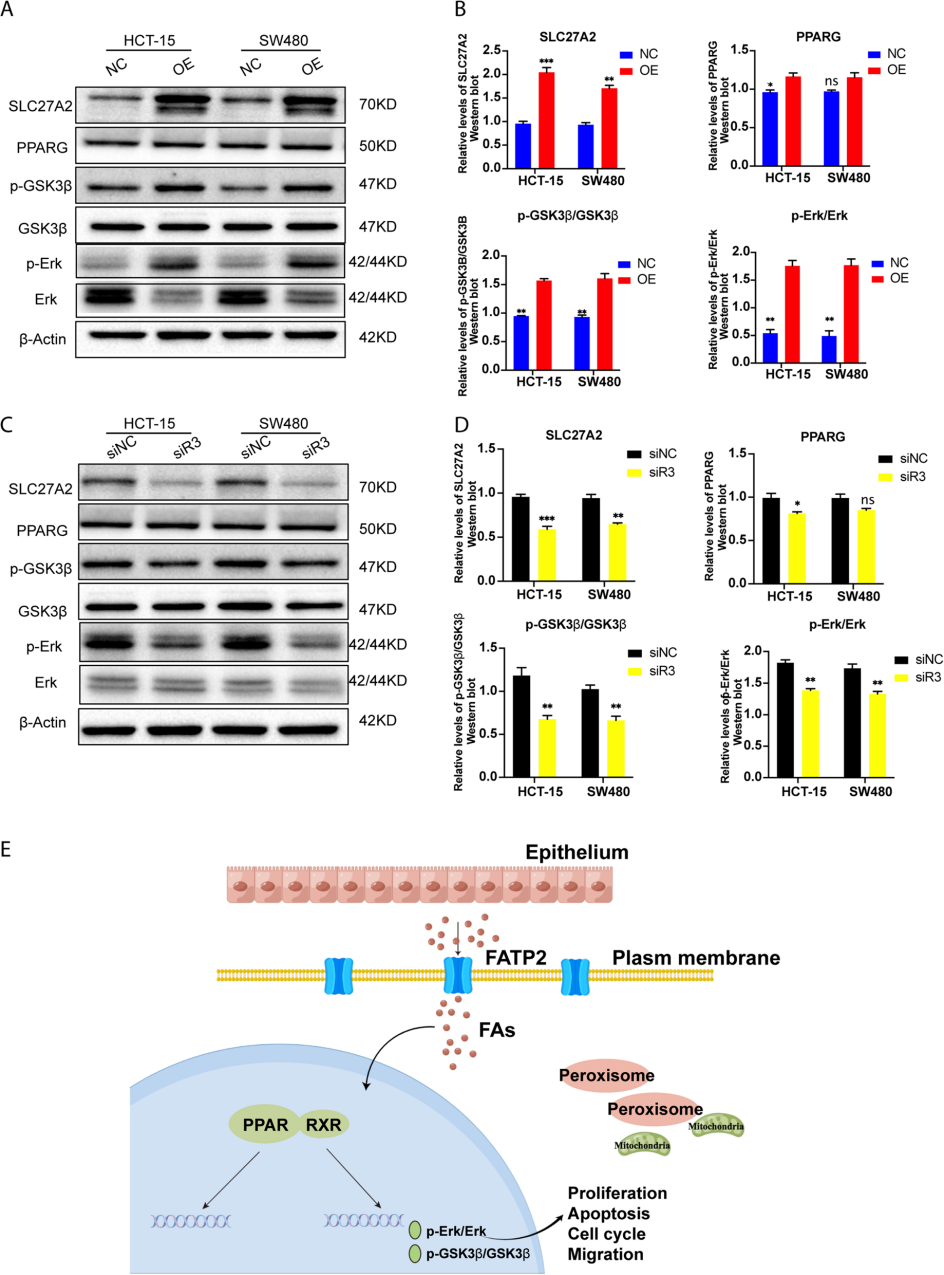
SLC27A2 reprogrammed colorectal cancer nongenic crosstalk regulation of PPARs. **A** The protein expression levels of SLC27A2, PPARG, p-GSK3β, GSK3β, p-Erk, and Erk when SLC27A2 was overexpressed. The grouping of blots cropped from diferent gels. The blots were cut prior to hybridisation with antibodies. The raw data with detail description and multiple exposure images was shown in Supplementary Fig. 1. The relative levels of SLC27A2, PPARG, p-GSK3β/GSK3β, and p-Erk/Erk in HCT-15 and SW480 between NC group and OE group. **C** The protein expression levels of SLC27A2, PPARG, p-GSK3β, GSK3β, p-Erk, and Erk when SLC27A2 wasknocked down. The grouping of blots cropped from diferent gels. The blots were cut prior to hybridisation with antibodies. The raw data with detail description and multiple exposure images was shown in Supplementary Fig. 1. **D** The relative levels of SLC27A2, PPARG, p-GSK3β/GSK3β, and p-Erk/Erk in HCT-15 and SW480 between siNC group and siR3 group. **E** The graphic abstract of SLC27A2 reprogramming colorectal cancer. SLC27A2: Solute carrier family 27 member 2; NC: negative control; OE: over expression; PPARG: Peroxisome proliferator activated receptor G; GSK3β:Glycogen Synthase Kinase 3 Beta; PPARG: Peroxisome proliferator activated receptor G; RXR: retinoid X receptor; FAs: Fatty acids; FATP2: Fatty acid transport protein 2

*PPARG* is a nuclear hormone receptor (NHR). Activated *PPARG* can regulate lipid homeostasis and metabolism [15]. We detected the the ratios of *p-Erk/Erk* and *p-GSK3β/GSK3β* when cells were transfected with plasmids or siRNAs to overexpress or knock down *SLC27A2* (encoding *FATP2*), respectively. Additionally, the efficiencies were verified by western blotting. The results showed that the ratios of *p-Erk/Erk* and *p-GSK3β/GSK3β* were elevated when *SLC27A2* was overexpressed (Fig. 5A ~ B). Conversely, the ratios were reduced when *SLC27A2* was knocked down (Fig. 5C ~ D). Non-genic crosstalk regulation of PPARs through *p-Erk/Erk* and *p-GSK3β/GSK3β* influenced FA metabolic reprogramming in CRC (Fig. 5E, Graphic abstract).

## Discussion

Peroxisome proliferator-activated receptors (*PPARs*) are ligand-activated nuclear hormone receptors (NHRs) and pivotal regulators in a series of lipid metabolic bioactivities, including adipocyte differentiation, lipid transportation and energy metabolism. *PPARs* isoforms can act as pro- or anti-tumorigenic factors. Preclinical or clinical evidence has proven that *PPAR* agonists or antagonists play critical roles in tumor metabolic reprogramming, cellular environmental homeostasis, and drug response [24, 25]. High-fat diet (HFD) was reported to contribute to CRC progression and liver metastasis, and *PPARD* antagonists could reverse this condition and might be beneficial for CRC treatment [26]. *PPARs* can induce FAO programming to maintain renewal of intestinal stem cells (ISCs) under HFD conditions [13]. In addition, HFD could also affect intestinal stem cell homeostasis through *PPARs*, *mTORC1*, *Wnt/GSK-3β*, or *PTEN* pathways [27]. Controversially, *PPARs* play different roles in tumor progression. In our previous study, we have demonstrated that *SLC27A2* regulated FA uptake and cell biological behavior in a metabolic manner in CRC cell lines. Previous studies revealed that *SLC27A2* regulated FAO to support ISC renewal [28], and mediated immune suppressive activity for myeloid-derived suppressor cells (MDSCs) in CRC mouse models [17]. We investigated the expression of *PPARs* and found differentially expressed levels in CRC. *PPARs* had a relatively close relationship with *SLC27A2* in CRC (Fig. 1). As important factors for proliferation, apoptosis and energy metabolism, the ratios of *p-Erk/Erk* and *p-GSK3β/GSK3β* varied when *SLC27A2* was overexpressed or knocked down via non-genic crosstalk regulation of *PPARG* (Fig. 5). Additionally, immune checkpoint inhibitors (ICIs) effective for CRC patients when the genetic phenotypes are mismatch-repair-deficient or microsatellite instability-high (dMMR/MSI-H) [29]. Recent studies have shown that *PPARG* induces programmed cell death ligand 1 (PD-L1) expression in CRC [30]. Encouragingly, targeting *PPARs* may be a new anti-tumor strategy.

The solute carrier protein (*SLC*) family is the second largest class of transmembrane transporters and is a potential drug target [31]. SLC27s (*SLC27A1* ~ *6*) are protein-encoding families involved in lipids metabolism. Fatty acid transport proteins (*FATP1* ~ *6*) play pivotal roles in fatty acid uptake and fatty acyl-CoA synthetase activity [32]. In this study, we found that *SLC27A2* was elevated in CRC (Fig. 1). Additionally, we have proved that *SLC27A2* played a critical role in biological behavior and was mechanically regulated via the FA metabolic pathway in CRC cell lines. Consistently, the expression of *SLC27A2* in colorectal cancer tissues was also higher to paired para-cancerous tissues in our ongoing study. The preliminary results indicated that knockdown of *SLC27A2* may reduce tumor burden in preclinical animal model. The differences between isolated cancer cells and paired normal colon cells from the models can be further analyzed by single-cell RNA sequencing (scRNA-seq), RNA sequencing (RNA-seq) or metabonomics analysis. By protein–protein interaction (PPI) network analysis, we found that *SLC27A2* had an obvious correlation with FAO–related genes (Fig. 2) and demonstrated that the mRNA expression levels of the genes were elevated when *SLC27A2* was overexpressed or reduced when *SLC27A2* was knocked down (Fig. 3). Considerable evidence has revealed that *SLC27A2* is related to various metabolic disorders or diseases, such as lipotoxicity, oxidative stress and energy production, nonalcoholic fatty liver disease (NAFLD), type 2 diabetes mellitus (T2DM), kidney fibrosis, and cancers [33, 34]. Additionally, *SLC27A2* regulated the function and number of peroxisomes in CRC (Fig. 4). Peroxisomes are metabolic organelles. Extensive studies have revealed the functional significance of peroxisomes, which are involved in FAO, cellular redox homeostasis, lipolysis and immunometabolism. The pathogenesis of cancer can be mediated by peroxisomes [35, 36]. *SLC27A2* could regulate cells peroxisomes and mitochondria FAO in melanoma cells to induce drug resistance [37]. In addition, *SLC27A2* regulated peroxisomes and mitochondria FAO to maintain ISC renewal [28]. Investigation of peroxisomes might provide new targeted therapeutic strategies. In our study, we explored the relationship between *SLC27A2* and FAO metabolic genes, and found *SLC27A2* could regulate FAO metabolic genes expression (Fig. 3). Metabolic reprogramming is a hallmark of malignant cells or the tumor microenvironment (TME), and cells adapt their metabolism to sustain biological processes [38]. Infiltrating immune cells play pivotal roles in the TME and coordinate immunosurveillance [39]. MDSCs mediates immune escape in cancer progression. *FATP2* was exclusively elevated in MDSCs and regulated the function of MDSCs via the lipid metabolic pathways [17]. Targeting *FATP2* could modulate lipid metabolism and reduce reactive oxygen species (ROS) production in MDSCs, thus enhancing ICIs efficacy [40]. In addition, *FATP2* regulated lipids metabolism in melanoma and induced resistance to targeted therapy. Inhibiting *FATP2* strongly overcame the phenotype [37]. *PPARs* regulate cancer cell progression through crosstalk with oncogenes or suppressor genes [41]. Previous study showed that *SLC39A1* impaired tumor metabolism and regulated ell proliferation, migration, and cell cycle through the *PPAR* crosstalk regulation in renal cell carcinoma (RCC) [42]. In our research, we investigated the crosstalk between *PPARs* and *SLC27A2*, and found non-genic crosstalk regulation of *PPARs* through *p-Erk/Erk* and *p-GSK3β/GSK3β* to influence FA metabolic reprogramming in CRC. Taken together, these findings might provide novel insights for cancer treatment. Targeting *SLC27A2/FATP2* or *PPARs* may identify new anti-tumor strategies, especially in metabolic therapy, immunotherapy, targeted therapy, immunometabolism or combinations.

## Conclusions

In our study, we verified that *SLC27A2* was overexpressed in CRC and that *SLC27A2* mediated FAO metabolism through non-genic crosstalk regulation of the *PPAR* pathway in CRC. Targeting metabolic reprogramming in cancers might provide new insights for anti-tumor strategies. Targeting *SLC27A2/FATP2* or *PPARs* might be a new strategy for cancer treatment.

## Supplementary Information

**Additional file 1: Supplementary Figure 1.** Original gels for all Western Blots in Figure 5 and Multiple exposure images.

**Additional file 2: Supplementary Figure 2.** The original ‘GEPIA2 Expression DIY on Box Plot’ mode and ‘GEPIA2 Correlation Analysis’ mode following the steps and we have clearly described in Materials and methods section in the manuscript.

**Additional file 3: Supplementary Table 1.** primers.

## Data Availability

The raw datas are available from the corresponding author on reasonable request. All datasets were public datasets and were freely available. The datasets generated and/or analyzed during the current study are available in the [GEPIA] repository (http://gepia2.cancer-pku.cn/#analysis, The raw data provided in Supplementary Fig. 2), and the [STRING] repository (https://cn.string-db.org/, adirect link: https://version-11-5.string-db.org/cgi/network?networkId=bMSHAa0TpJwp; direct link: https://version-11-5.string-db.org/cgi/network?networkId=bq05befWQUz1;) and Graphic Abstract was performed on Figdraw (https://www.figdraw.com/static/index.html#/, accession numbers: 788566346027118592; Figure ID:UWYWTc1d3c).

## Abbreviations

*PPARs*: Peroxisome proliferator activated receptors
NHRs: Nuclear hormone receptors
FA: Fatty acid
*SLC27A2*: Solute carrier family 27 member 2
CRC: Colorectal cancer
PPI: Protein–protein interaction
FAO: Fatty acid oxidation
*ABCD3*: ATP Binding Cassette Subfamily D Member 3
TME: Tumor microenvironment
*FAT*: Fatty acid translocase
*FABPs*: Fatty acid binding proteins
*FATPs*: Fatty acid transport proteins
ISCs: Intestinal stem cells
HFD: High-fat diet
RXR: Retinoid X receptor
PPREs: Peroxisome proliferator response elements
FBS: Fetal bovine serum
PA: Palmitic acid
ORO: Oil-Red-O
RT: Room temperature
TBST: Tris Buffered Saline with Tween-20
qRT‒PCR: Quantitative reverse transcriptase-PCR
TCGA: The Cancer Genome Atlas
GTEx: Genotype-Tissue Expression
GEPIA: Gene Expression Profiling Interactive Analysis
*FATP2*: Fatty acid transport protein 2
MDSCs: Myeloid-derived suppressor cells
ICIs: Immune checkpoint inhibitors
dMMR: Mismatch-repair-deficient
MSI-H: Microsatellite instability-high
*PD-L1*: Programmed cell death ligand 1
scRNA-seq: Single-cell RNA sequencing
RNA-seq: RNA sequencing
NAFLD: Non-alcoholic fatty liver disease
T2DM: Type 2 diabetes mellitus
ROS: Reactive oxygen species
*SLC39A1*: Solute carrier family 39 member 1
RCC: Renal cell carcinoma
NC: Negative control
OE: Over expression
