# Supplementary material for: SLC27A2 mediates FAO in colorectal cancer through nongenic crosstalk regulation of the PPARs pathway
Source: BMC Cancer. 2023 Apr 11;23:335. doi: 10.1186/s12885-023-10816-3 (PMC10091540; doi:10.1186/s12885-023-10816-3)
Supplement: Supplementary file 1 — Additional file 1: Supplementary Figure 1. Original gels for all Western Blots in Figure 5 and Multiple exposure images. [file 12885_2023_10816_MOESM1_ESM.pdf]

Supplementary Figure 1: Original gels for all Western Blots in Figure 5 and Multiple exposure images

The original Western Blots in Figure 5 are presented below and original images are clearly labelled. Methods and figure legends for these blots have been clearly described in the manuscript.

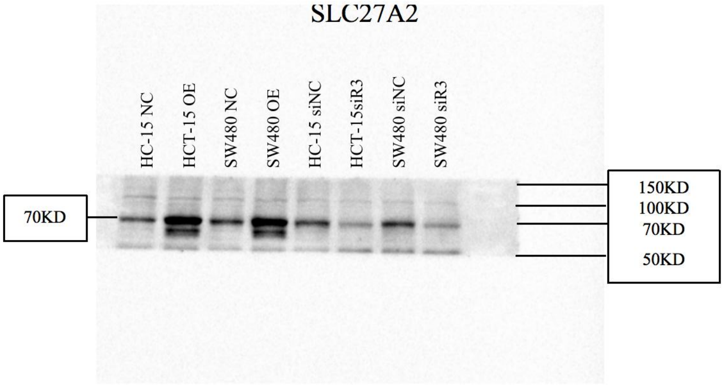

Figure5 S) The original Western Blot images of Protein SLC27A2 in Fig. 5A and Fig. 5C in the manuscript. From left to right: HC-15 NC, HCT-15 OE, SW480 NC, SW480 OE, HC-15 siNC, HCT-15 siR3, SW480 siNC, SW480 siR3. The expected molecular weight is 70KD.

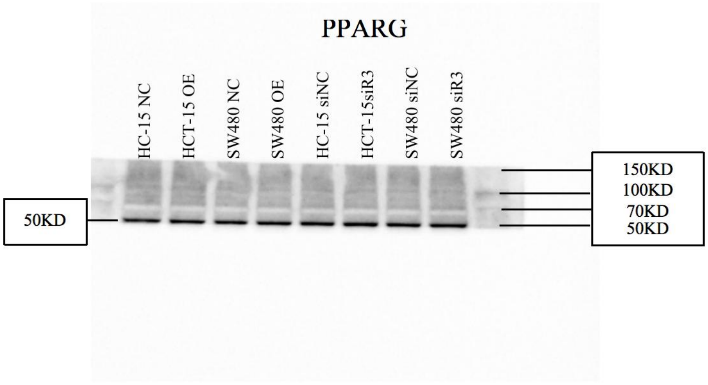

Figure5 S) The original Western Blot images of Protein PPARG in in Fig. 5A and Fig. 5C in the manuscript. From left to right: HC-15 NC, HCT-15 OE, SW480 NC, SW480 OE, HC-15 siNC, HCT-15 siR3, SW480 siNC, SW480 siR3. The expected molecular weight is 50KD.

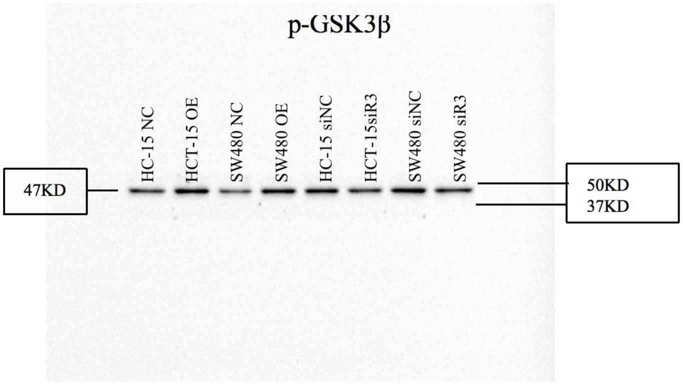

Figure5 S) The original Western Blot images of Protein p-GSK3β in Fig. 5A and Fig. 5C in the manuscript. From left to right: HC-15 NC, HCT-15 OE, SW480 NC, SW480 OE, HC-15 siNC, HCT-15 siR3, SW480 siNC, SW480 siR3. The expected molecular weight is 47KD.

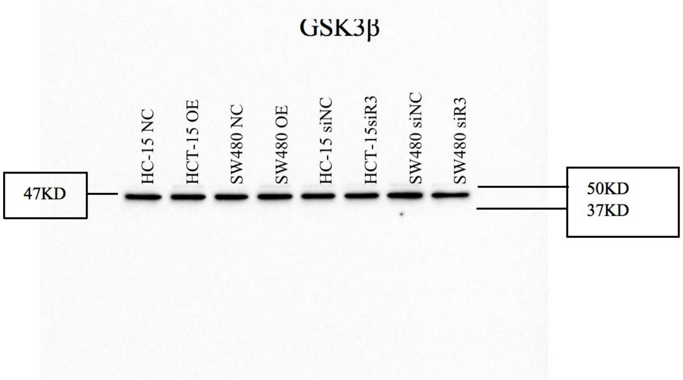

Figure5 S) The original Western Blot images of Protein GSK3β in Fig. 5A and Fig. 5C in the manuscript. From left to right: HC-15 NC, HCT-15 OE, SW480 NC, SW480 OE, HC-15 siNC, HCT-15 siR3, SW480 siNC, SW480 siR3. The expected molecular weight is 47KD.

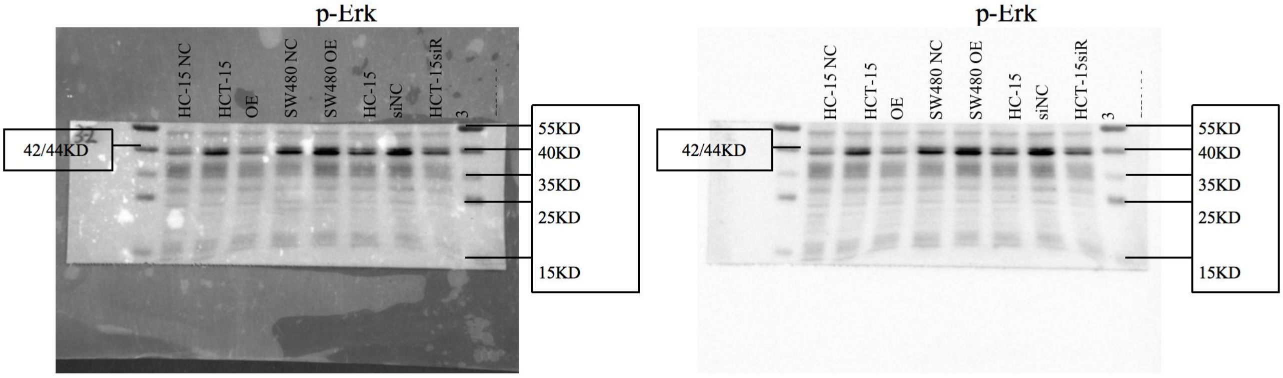

Figure5 S) The original Western Blot images of Protein p-Erk in Fig. 5A and Fig. 5C in the manuscript. From left to right: HC-15 NC, HCT-15 OE, SW480 NC, SW480 OE, HC-15 siNC, HCT-15 siR3, SW480 siNC, SW480 siR3. The expected molecular weight is 42/44KD.

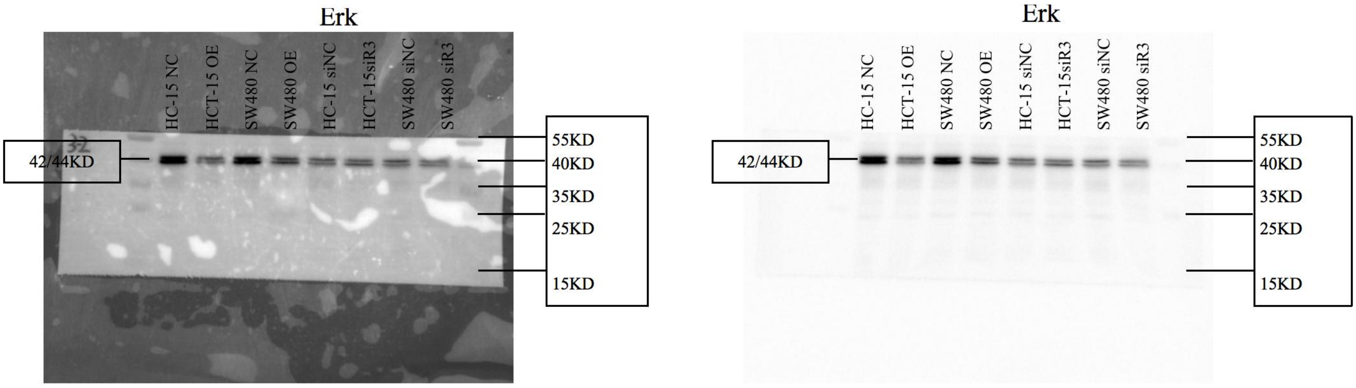

Figure5 S) The original Western Blot images of Protein Erk in Fig. 5A and Fig. 5C in the manuscript. From left to right: HC-15 NC, HCT-15 OE, SW480 NC, SW480 OE, HC-15 siNC, HCT-15 siR3, SW480 siNC, SW480 siR3. The expected molecular weight is 42/44KD.

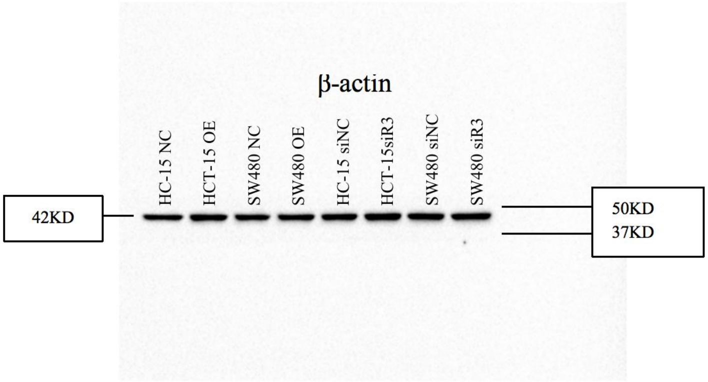

Figure5 S) The original Western Blot images of Protein β-actin in Fig. 5A and Fig. 5C in the manuscript. From left to right: HC-15 NC, HCT-15 OE, SW480 NC, SW480 OE, HC-15 siNC, HCT-15 siR3, SW480 siNC, SW480 siR3. The expected molecular weight is 42KD.

Multiple exposure images

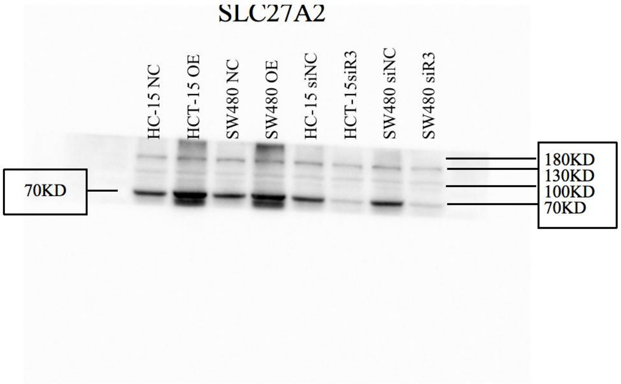

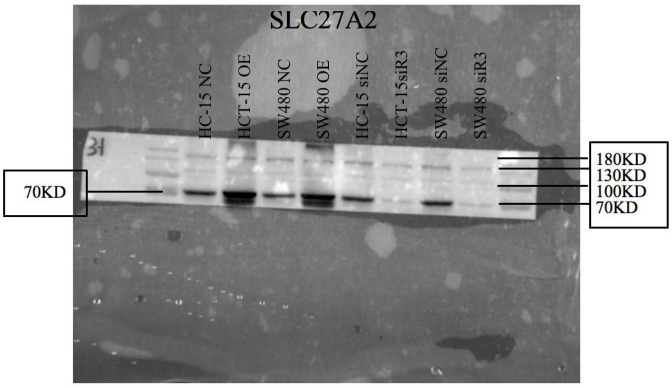

The original Western Blot images of Protein SLC27A2. From left to right: Marker, HC-15 NC, HCT-15 OE, SW480 NC, SW480 OE, HC-15 siNC, HCT-15 siR3, SW480 siNC, SW480 siR3, Marker. The expected molecular weight is 70KD.

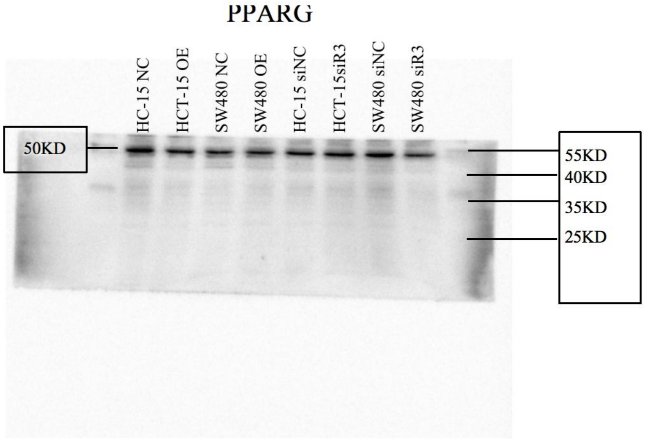

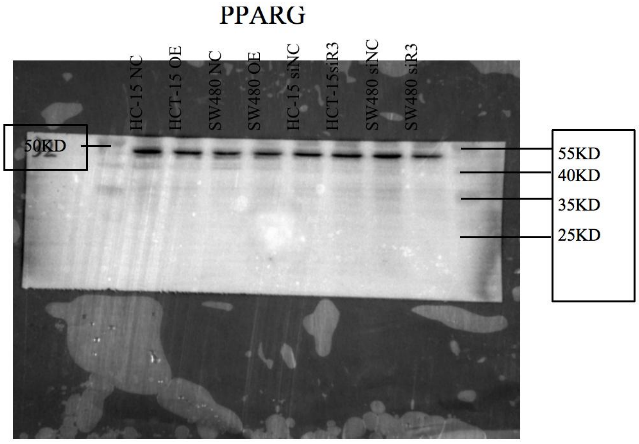

The original Western Blot images of Protein PPARG. From left to right: Marker, HC-15 NC, HCT-15 OE, SW480 NC, SW480 OE, HC-15 siNC, HCT-15 siR3, SW480 siNC, SW480 siR3, Marker. The expected molecular weight is 50KD.

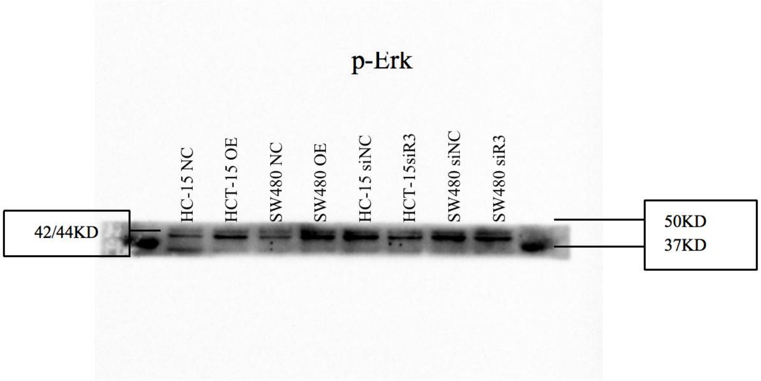

The original Western Blot images of Protein p-Erk. From left to right: Marker, HC-15 NC, HCT-15 OE, SW480 NC, SW480 OE, HC-15 siNC, HCT-15 siR3, SW480 siNC, SW480 siR3, Marker. The expected molecular weight is 42/44KD.

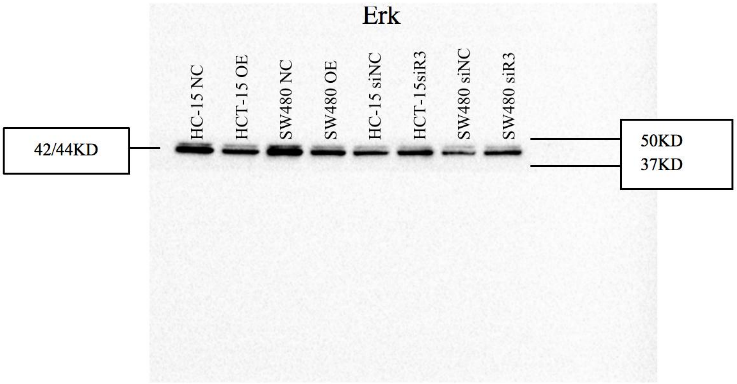

The original Western Blot images of Protein Erk. From left to right: Marker, HC-15 NC, HCT-15 OE, SW480 NC, SW480 OE, HC-15 siNC, HCT-15 siR3, SW480 siNC, SW480 siR3, Marker. The expected molecular weight is 42/44KD.

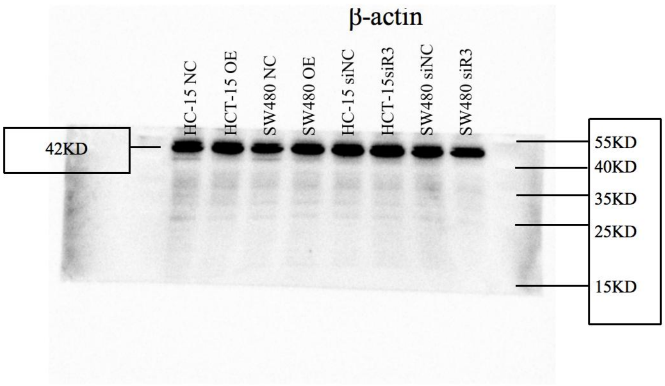

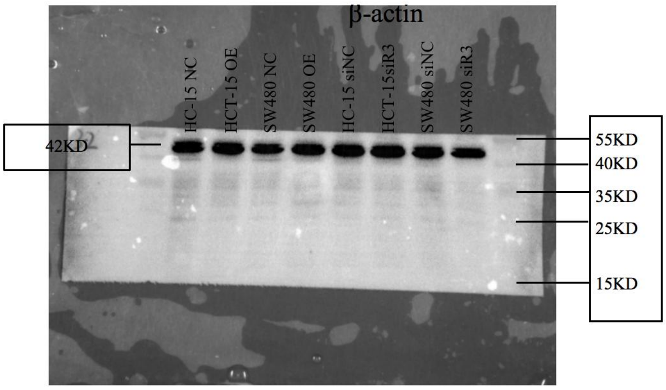

The original Western Blot images of Protein β-actin. From left to right: Marker, HC-15 NC, HCT-15 OE, SW480 NC, SW480 OE, HC-15 siNC, HCT-15 siR3, SW480 siNC, SW480 siR3, Marker. The expected molecular weight is 42KD.

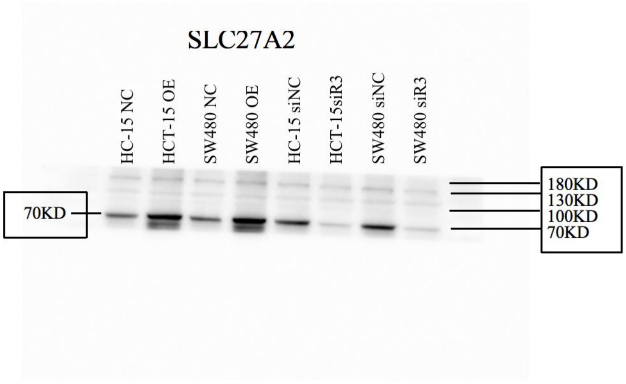

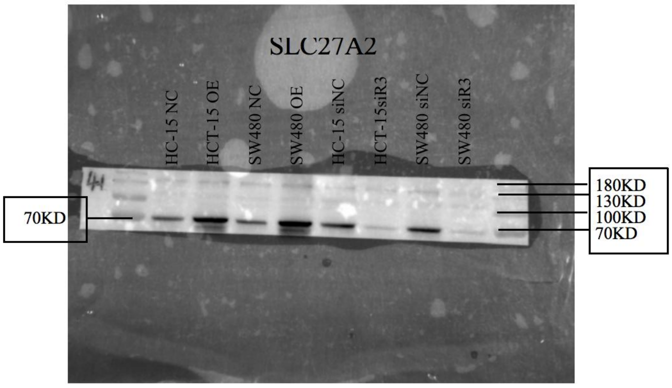

The original Western Blot images of Protein SLC27A2. From left to right: Marker, HC-15 NC, HCT-15 OE, SW480 NC, SW480 OE, HC-15 siNC, HCT-15 siR3, SW480 siNC, SW480 siR3, Marker. The expected molecular weight is 70KD.

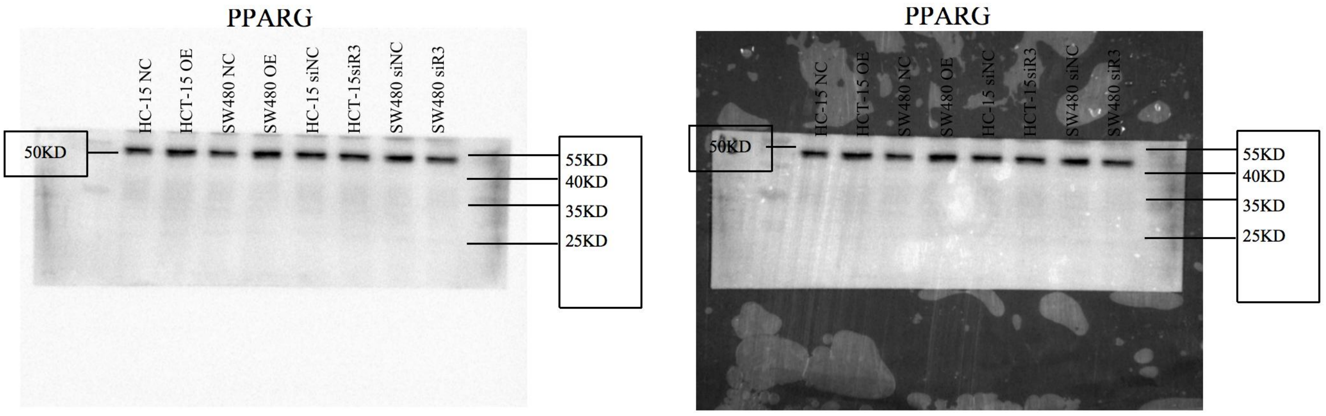

The original Western Blot images of Protein PPARG. From left to right: Marker, HC-15 NC, HCT-15 OE, SW480 NC, SW480 OE, HC-15 siNC, HCT-15 siR3, SW480 siNC, SW480 siR3, Marker. The expected molecular weight is 50KD.

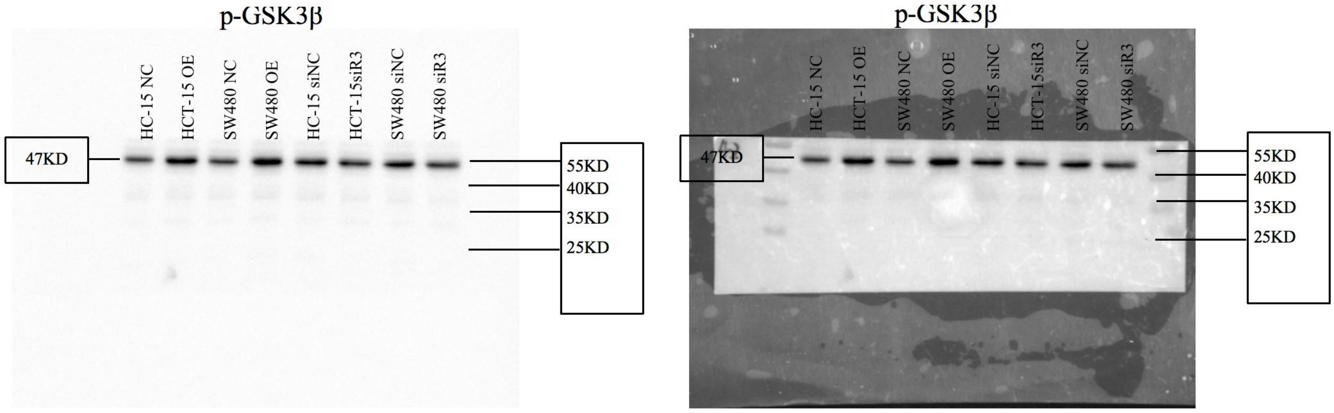

The original Western Blot images of Protein p-GSK3β. From left to right: Marker, HC-15 NC, HCT-15 OE, SW480 NC, SW480 OE, HC-15 siNC, HCT-15 siR3, SW480 siNC, SW480 siR3, Marker. The expected molecular weight is 47KD.

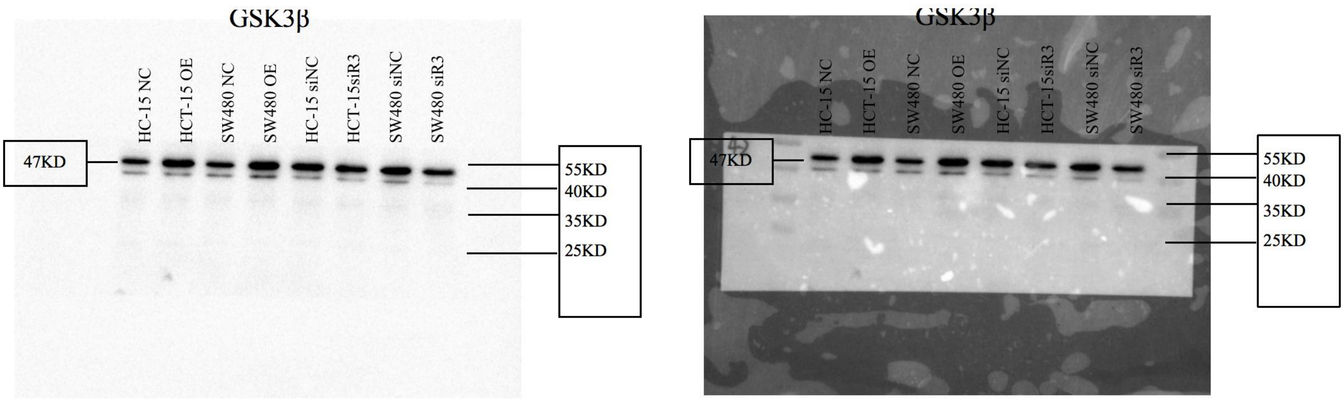

The original Western Blot images of Protein GSK3β. From left to right: Marker, HC-15 NC, HCT-15 OE, SW480 NC, SW480 OE, HC-15 siNC, HCT-15 siR3, SW480 siNC, SW480 siR3, Marker. The expected molecular weight is 47KD.

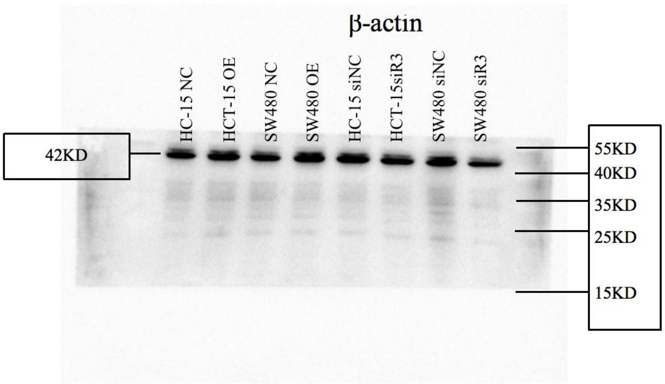

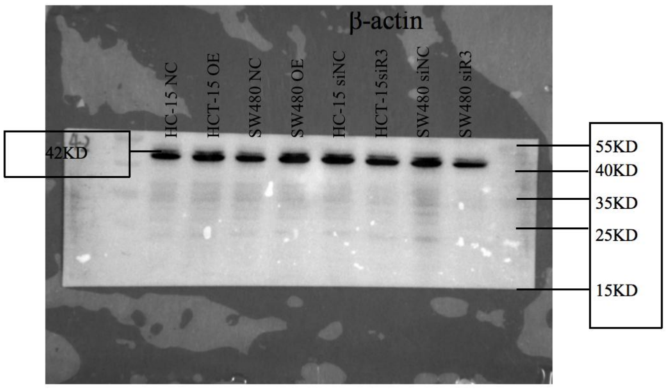

The original Western Blot images of Protein β-actin. From left to right: Marker, HC-15 NC, HCT-15 OE, SW480 NC, SW480 OE, HC-15 siNC, HCT-15 siR3, SW480 siNC, SW480 siR3, Marker. The expected molecular weight is 42KD.

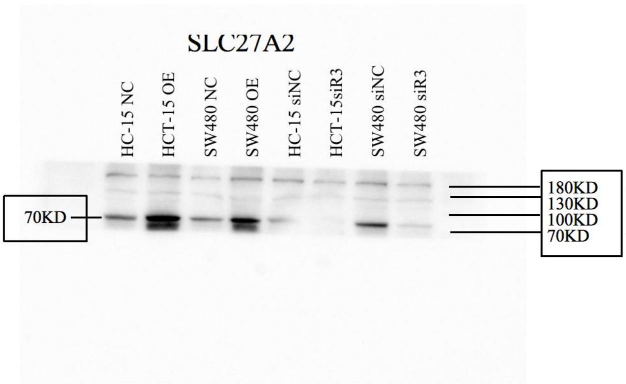

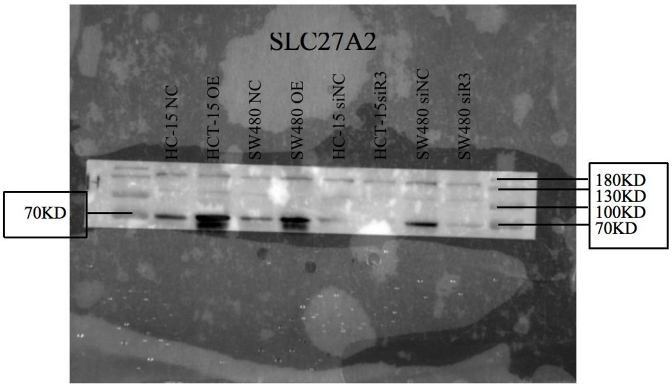

The original Western Blot images of Protein SLC27A2. From left to right: Marker, HC-15 NC, HCT-15 OE, SW480 NC, SW480 OE, HC-15 siNC, HCT-15 siR3, SW480 siNC, SW480 siR3, Marker. The expected molecular weight is 70KD.

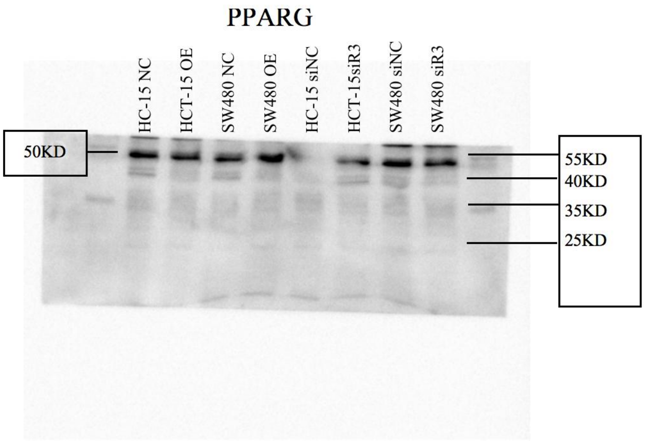

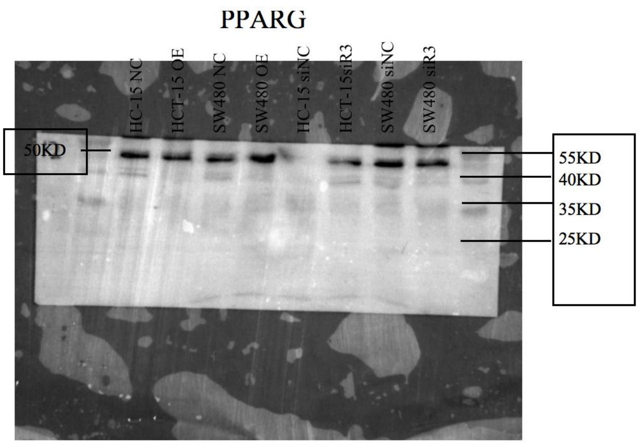

The original Western Blot images of Protein PPARG. From left to right: Marker, HC-15 NC, HCT-15 OE, SW480 NC, SW480 OE, HC-15 siNC, HCT-15 siR3, SW480 siNC, SW480 siR3, Marker. The expected molecular weight is 50KD.

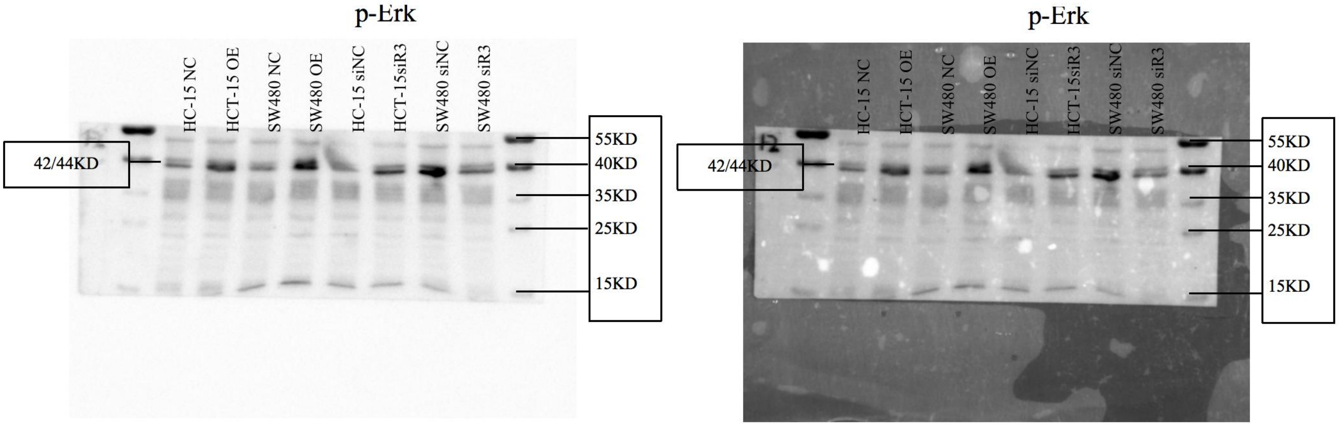

The original Western Blot images of Protein p-Erk. From left to right: Marker, HC-15 NC, HCT-15 OE, SW480 NC, SW480 OE, HC-15 siNC, HCT-15 siR3, SW480 siNC, SW480 siR3, Marker. The expected molecular weight is 42/44KD.

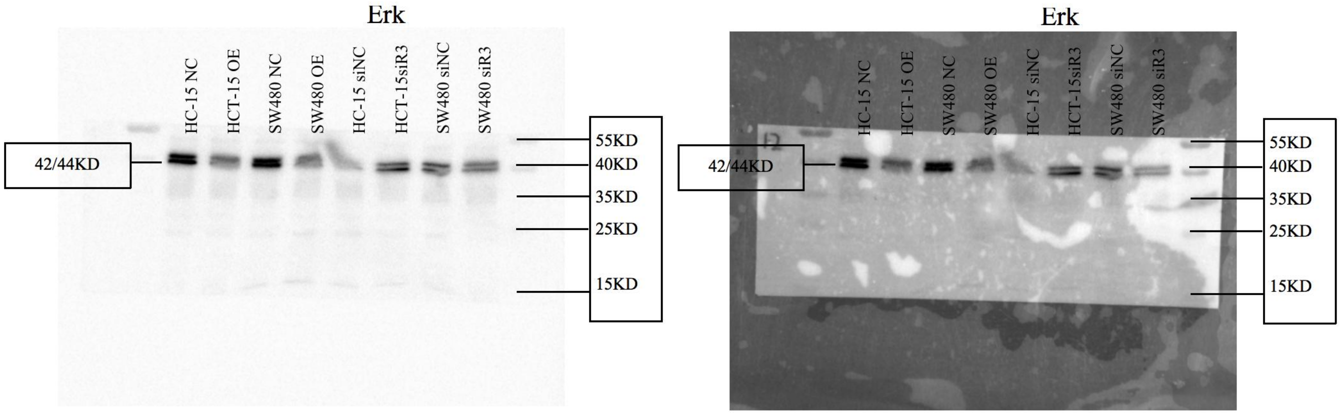

The original Western Blot images of Protein Erk. From left to right: Marker, HC-15 NC, HCT-15 OE, SW480 NC, SW480 OE, HC-15 siNC, HCT-15 siR3, SW480 siNC, SW480 siR3, Marker. The expected molecular weight is 42/44KD.

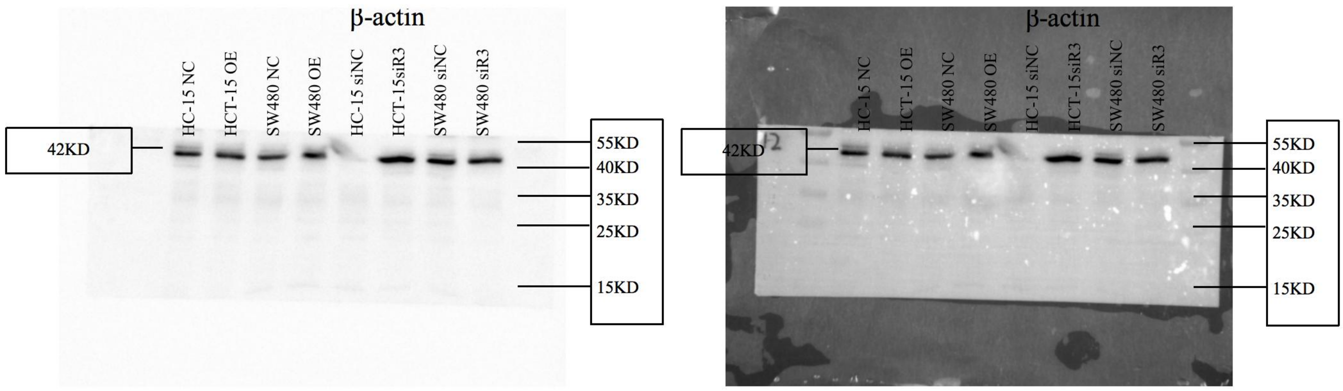

The original Western Blot images of Protein β-actin. From left to right: Marker, HC-15 NC, HCT-15 OE, SW480 NC, SW480 OE, HC-15 siNC, HCT-15 siR3, SW480 siNC, SW480 siR3, Marker. The expected molecular weight is 42KD.

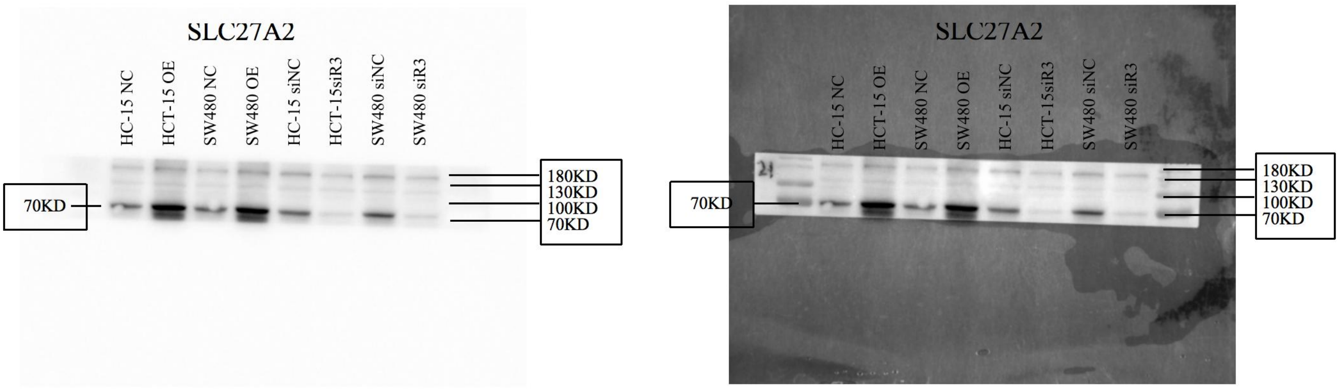

The original Western Blot images of Protein SLC27A2. From left to right: Marker, HC-15 NC, HCT-15 OE, SW480 NC, SW480 OE, HC-15 siNC, HCT-15 siR3, SW480 siNC, SW480 siR3, Marker. The expected molecular weight is 70KD.

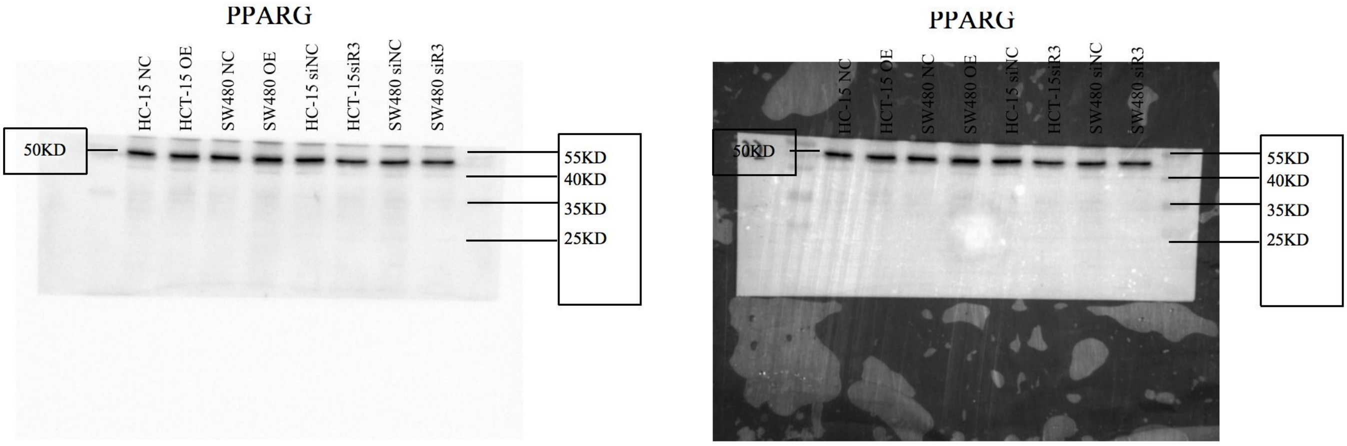

The original Western blot images of Protein PPARG. From left to right: Marker, HC-15 NC, HCT-15 OE, SW480 NC, SW480 OE, HC-15 siNC, HCT-15 siR3, SW480 siNC, SW480 siR3, Marker. The expected molecular weight is 50KD.

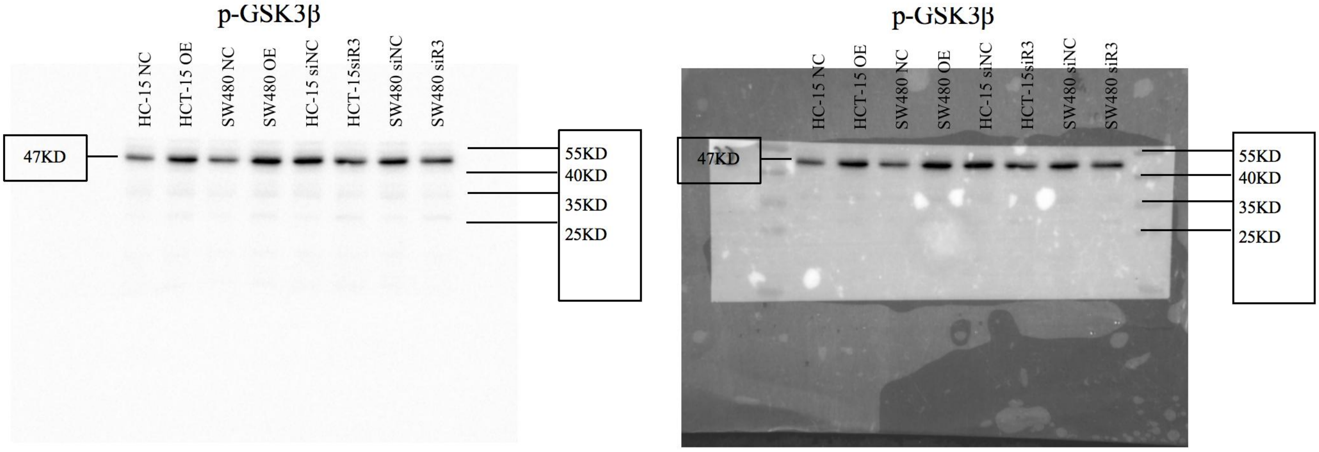

The original Western Blot images of Protein p-GSK3β. From left to right: Marker, HC-15 NC, HCT-15 OE, SW480 NC, SW480 OE, HC-15 siNC, HCT-15 siR3, SW480 siNC, SW480 siR3, Marker. The expected molecular weight is 47KD.

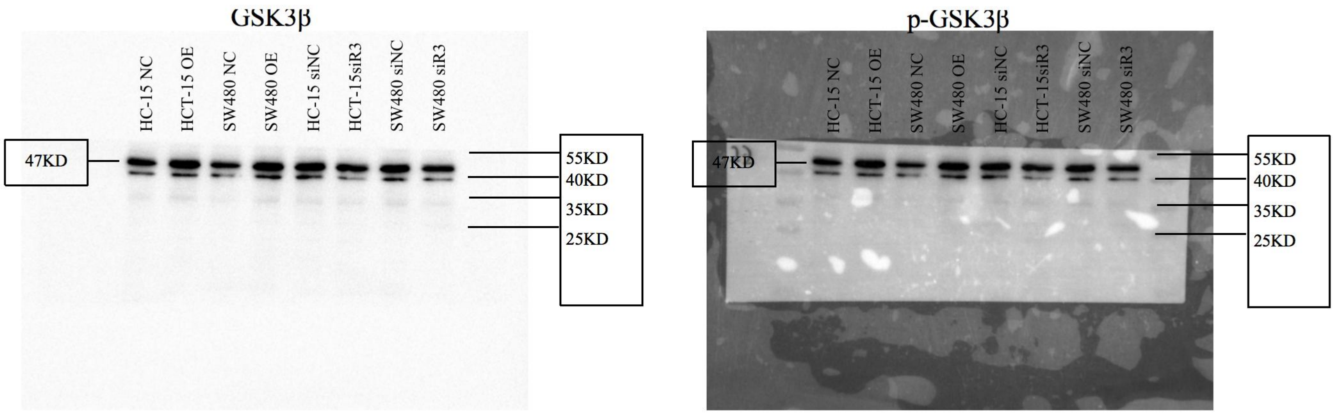

The original Western Blot images of Protein GSK3β. From left to right: Marker, HC-15 NC, HCT-15 OE, SW480 NC, SW480 OE, HC-15 siNC, HCT-15 siR3, SW480 siNC, SW480 siR3, Marker. The expected molecular weight is 47KD.

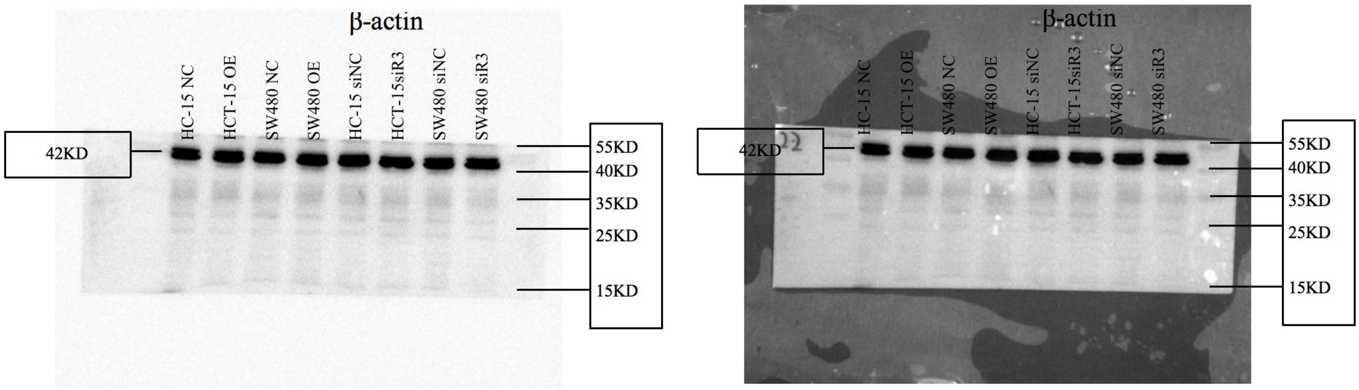

The original Western Blot images of Protein β-actin. From left to right: Marker, HC-15 NC, HCT-15 OE, SW480 NC, SW480 OE, HC-15 siNC, HCT-15 siR3, SW480 siNC, SW480 siR3, Marker. The expected molecular weight is 42KD.
