# Supplementary material for: SLC27A2 mediates FAO in colorectal cancer through nongenic crosstalk regulation of the PPARs pathway
Source: BMC Cancer. 2023 Apr 11;23:335. doi: 10.1186/s12885-023-10816-3 (PMC10091540; doi:10.1186/s12885-023-10816-3)
Supplement: Supplementary file 2 — Additional file 2: Supplementary Figure 2. The original ‘GEPIA2 Expression DIY on Box Plot’ mode and ‘GEPIA2 Correlation Analysis’ mode following the steps and we have clearly described in Materials and methods section in the manuscript. [file 12885_2023_10816_MOESM2_ESM.pdf]

Supplementary Figure2

The original ‘GEPIA2 Expression DIY on Box Plot’ mode and ‘GEPIA2 Correlation Analysis’ mode following the steps and we have clearly described in Materials and methods section in the manuscript.

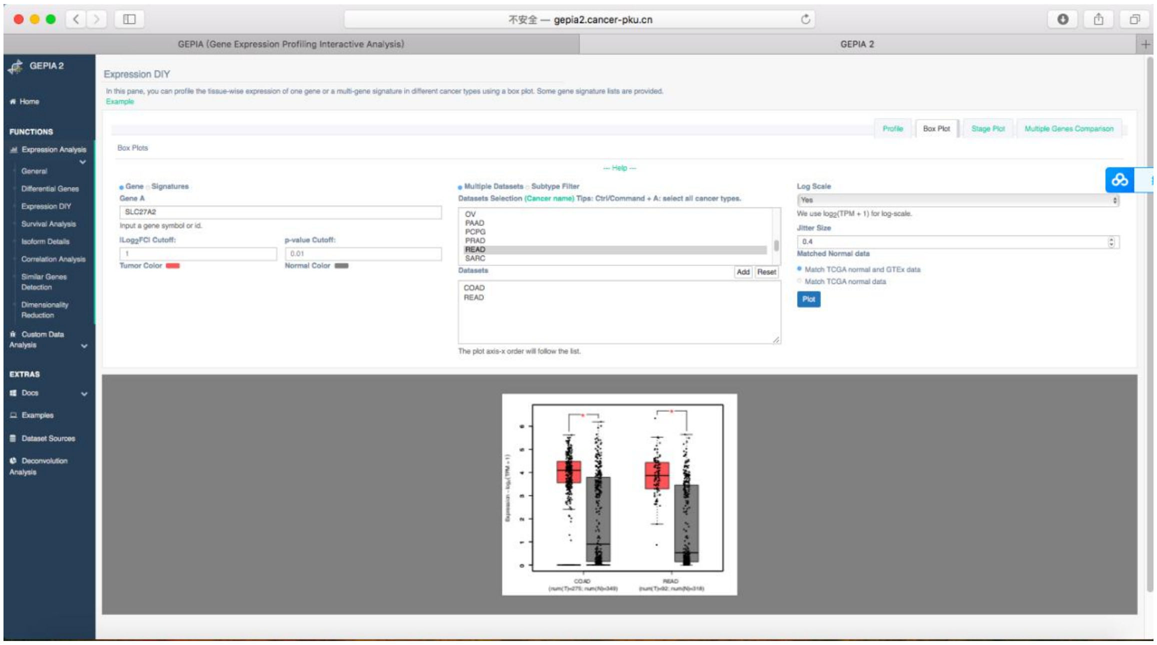

The original ‘GEPIA2 Expression DIY on Box Plot’ mode of SLC27A2 expression in COAD or READ in Fig1.A, and we provided the data in Supplementary data 1.

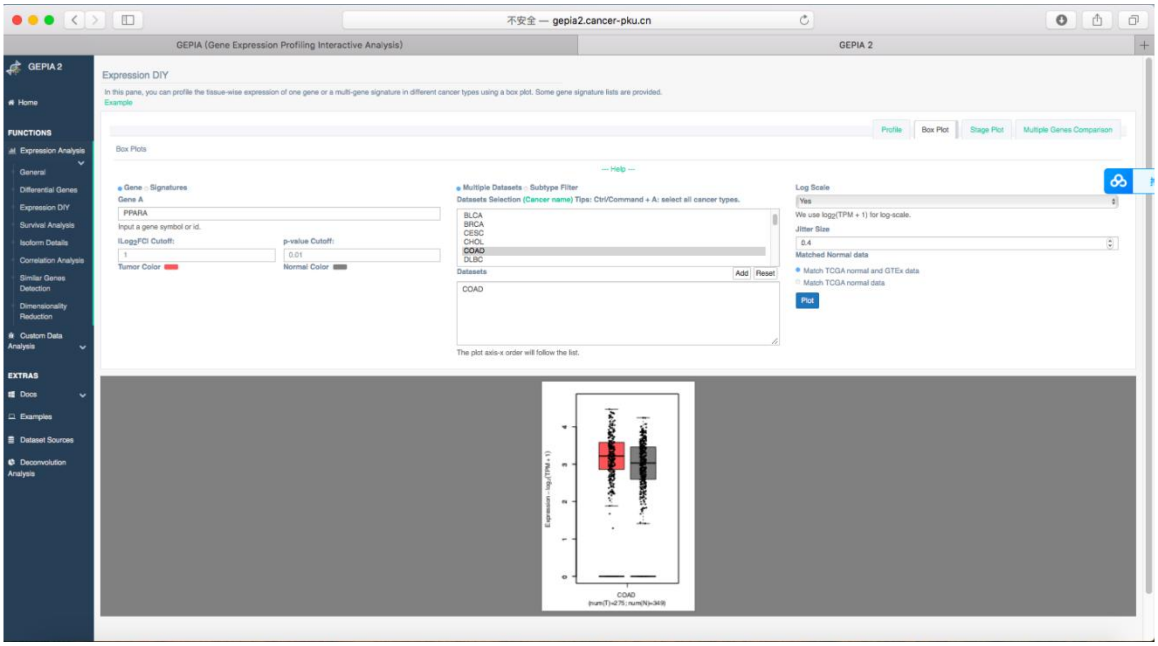

The original ‘GEPIA2 Expression DIY on Box Plot’ mode of PPARA expression in COAD in Fig1.B, and we provided the data in Supplementary data 2.

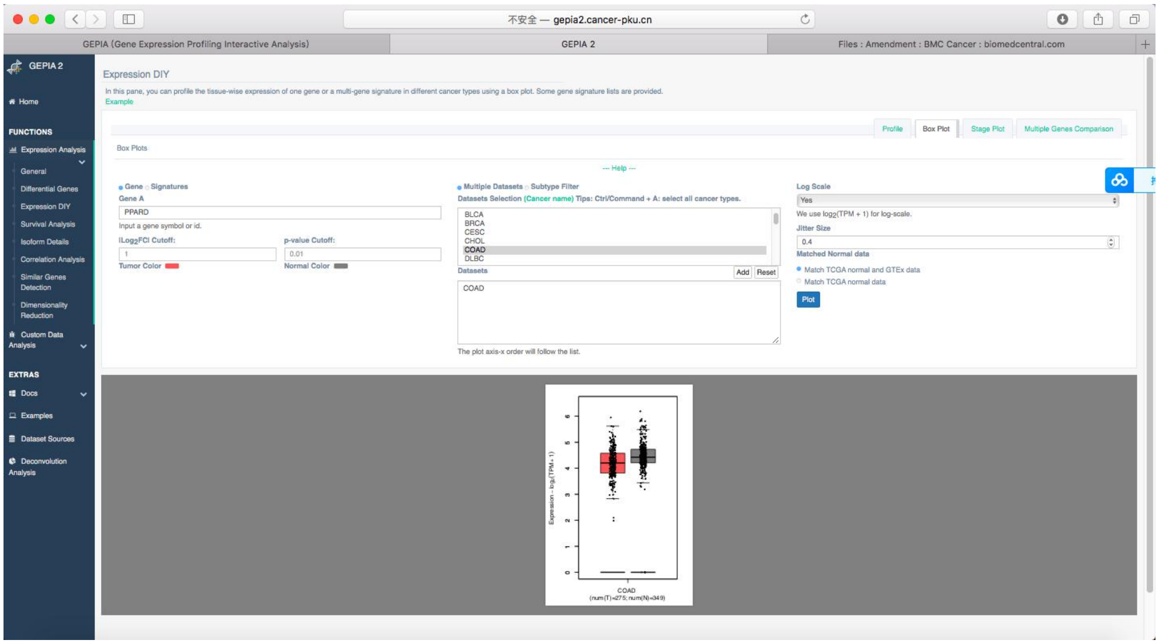

The original ‘GEPIA2 Expression DIY on Box Plot’ mode of PPARD expression in COAD in Fig1.B, and we provided the data in Supplementary data 3.

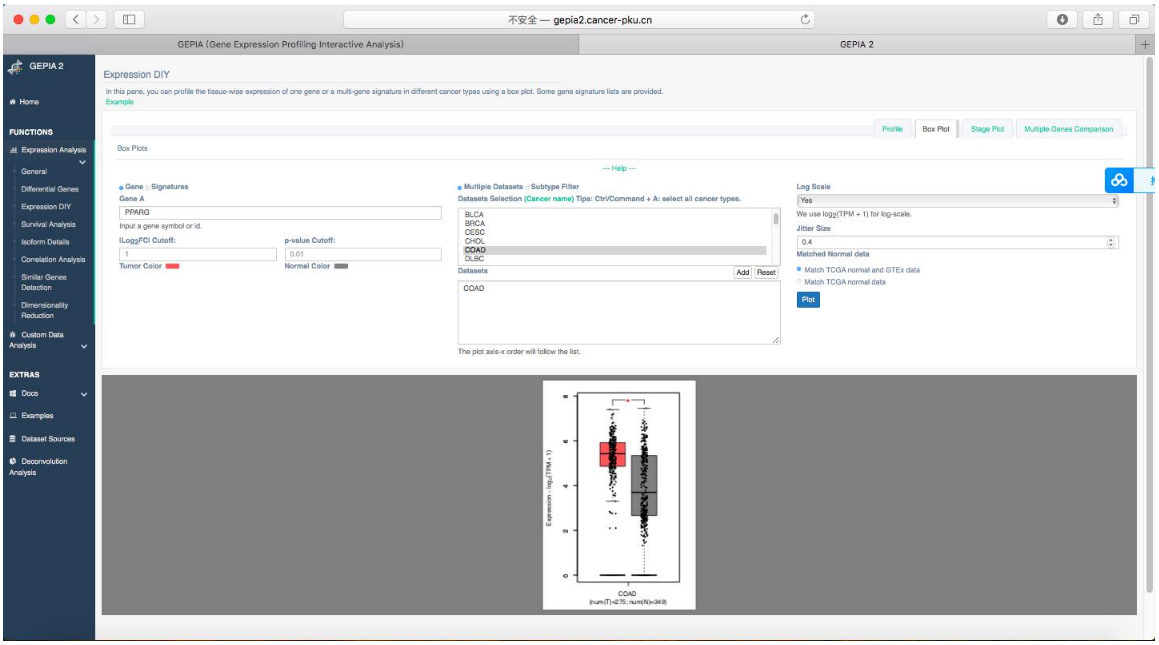

The original ‘GEPIA2 Expression DIY on Box Plot’ mode of PPARG expression in COAD in Fig1.B, and we provided the data in Supplementary data 4.

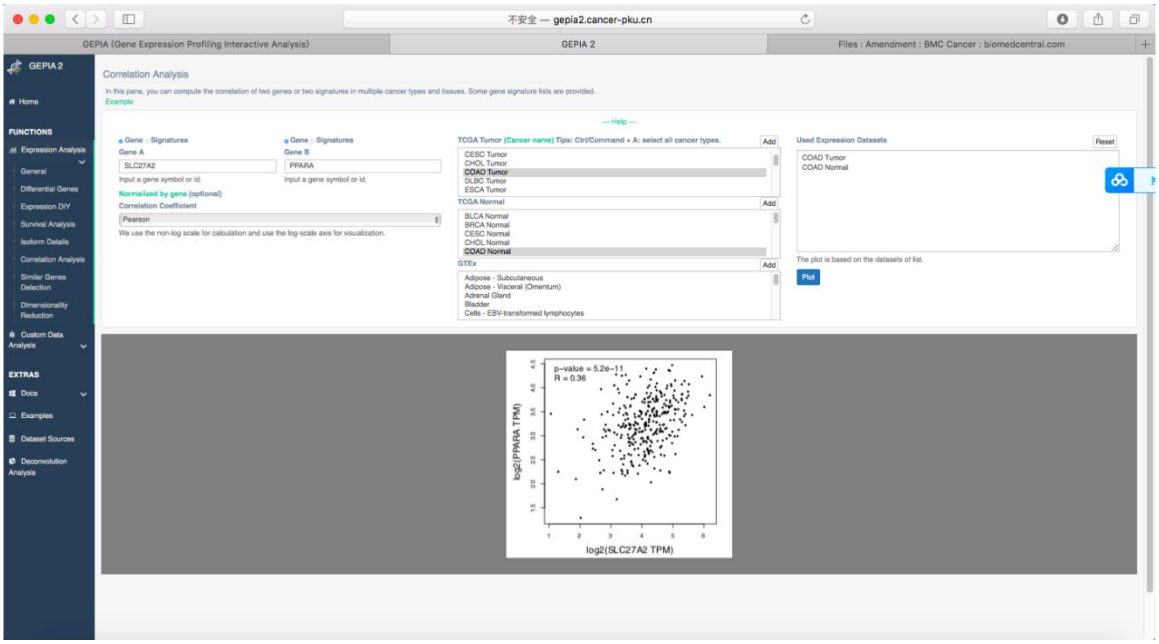

The original 'GEPIA2 Correlation Analysis' mode of the correlation of SLC27A2 with PPARA in COAD in Fig1.C, and we provided the data in Supplementary data 5.

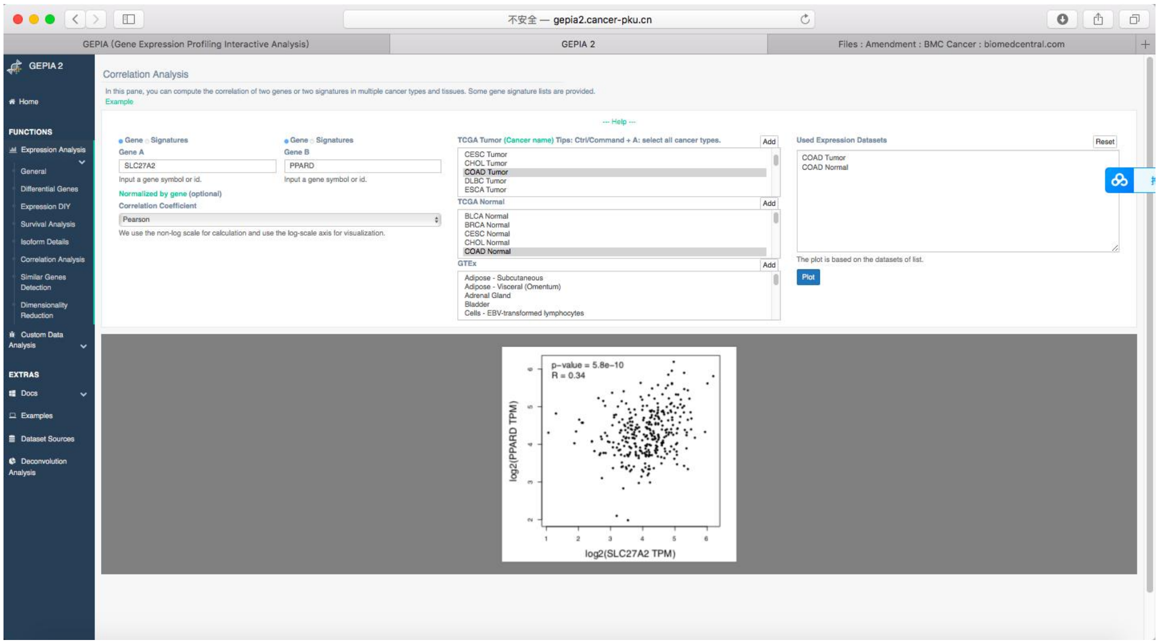

The original 'GEPIA2 Correlation Analysis' mode of the correlation of SLC27A2 with PPARD in COAD in Fig1.C, and we provided the data in Supplementary data 6.

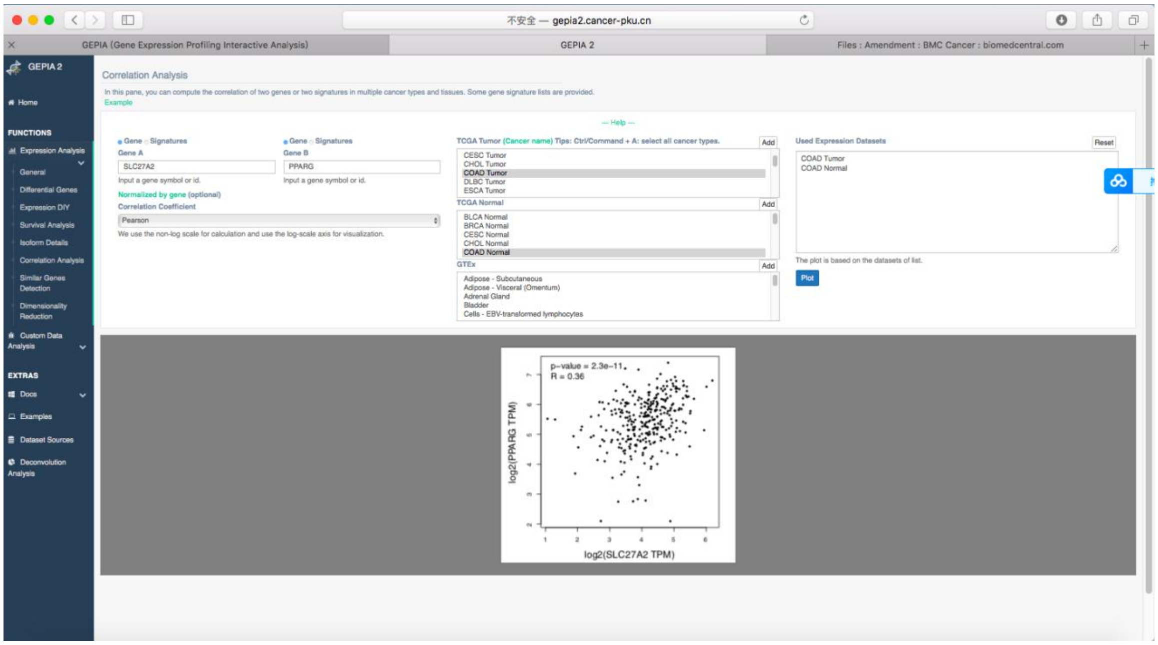

The original 'GEPIA2 Correlation Analysis' mode of the correlation of SLC27A2 with PPARG in COAD in Fig1.C, and we provided the data in Supplementary data 7.
